# The effect of ankle-foot orthoses on fall/near fall incidence in patients with (sub-)acute stroke: A randomized controlled trial

**DOI:** 10.1371/journal.pone.0213538

**Published:** 2019-03-12

**Authors:** Corien D. M. Nikamp, Marte S. H. Hobbelink, Job van der Palen, Hermie J. Hermens, Johan S. Rietman, Jaap H. Buurke

**Affiliations:** 1 Roessingh Research and Development, Enschede, the Netherlands; 2 Department of Biomedical Signals and Systems, Technical Medical Centre, University of Twente, Enschede, the Netherlands; 3 Medisch Spectrum Twente, Medical School Twente, Enschede, the Netherlands; 4 Department of Research Methodology, Measurement and Data Analysis, University of Twente, Enschede, the Netherlands; 5 Department of Biomechanical Engineering, Technical Medical Centre, University of Twente, Enschede, the Netherlands; 6 Roessingh Center for Rehabilitation, Enschede, the Netherlands; Universitat de Valencia, SPAIN

## Abstract

Falls are commonly reported post-stroke. Ankle-foot orthoses (AFOs) are often provided to improve safety and walking, but the effect of their use in the reduction of falls after stroke is unknown. A randomized controlled trial (RCT) on the effects of AFO-provision after stroke was performed. Effects on clinical scales, 3D-gait kinematics and muscle-activity were previously reported. This paper aims to study the effects of AFO-provision on occurrence and circumstances of falls/near falls. The RCT included unilateral hemiparetic stroke patients. AFOs were provided either early (study week 1) or delayed (study week 9). Both groups were compared in the first eight weeks of the study and diaries were used to register falls/near falls and their circumstances. Follow-up measurements were performed in week 9–52, in which both groups were provided with AFOs. Functional Ambulation Categories and Berg Balance Scale were assessed to determine walking independence and balance, respectively. Last known scores were noted in case of an incident. Thirty-three subjects were included (16 early, 17 delayed). In week 1–8, the early group, who were provided with AFOs, fell significantly more frequently compared with the delayed group, 11 versus 4 times, respectively (Incidence Rate Ratio = 2.9, *p* = 0.039). Out of the falls recorded in the early group, 63.6% occurred without wearing AFOs. Most of these falls occurred during transfers (36.4%) and standing (27.3%), and notably it were the subjects who did not have independent walking ability. No differences were found for near falls in week 1–8, or for falls/near falls in week 9–52. Six severe consequences (including fractures) were reported from a fall. To conclude, the subjects provided with AFOs early after stroke reported a higher number of falls, compared to the subjects that had not yet been provided with AFOs. However, in the subjects provided with AFOs, 63.6% of the falls occurred whilst without wearing the AFO. Furthermore, the majority of these incidents took place whilst subjects had no independent walking ability. This raises an interesting question of the importance of careful instructions to patients and their relatives, and the influence of potential cognitive impairments on the ability of the subjects to take on these instructions.

## Introduction

Loss of postural control is a major health problem in patients after stroke [1]. Delayed muscle, kinetic and kinematic responses to external perturbations are found [2]. Together with reduced postural stability during quiet standing, these delayed and less coordinated responses to both self-induced and external balance perturbations are reported as risk factors for falls [2]. In addition, walking ability is often affected after stroke. Insufficient foot clearance because of decreased flexion of the affected lower limb increases the risk for stumbling and falls [2]. Furthermore, depression, hemineglect, sensory and cognitive deficits have been explored and are important fall risk factors [2, 3]. As a result of one or more of these factors, falls occur frequently [4]. Studies show that after stroke, between 14%-65% of patients fall at least once during hospitalization, and between 37%-73% fall during the first six months following discharge from hospital [3]. The high risk of falls is present in all stages of rehabilitation, but higher fall rates are found immediately after discharge from hospital or rehabilitation clinic [2]. The number of falls that are reported varies widely between studies, with fall incidence rates varying between 1.3–6.5 falls each person-year [2]. Noteworthy is that these rates are much higher than the incidence rates of ~0.65 falls each person-year which have been documented in the general population of older people [5]. Soft tissue injuries are the most common injury from a fall [2, 3]. Serious injuries such as fractures are also reported, with numbers ranging between 0.6%-8.5% [2] and 1%-15% [3]. In addition to the physical consequences, falls often result in fear of falling, which in turn is related to balance and gait deficits and reduced physical activity and deconditioning [2]. Fear of falling is found in many patients who fall (88%) [6], this concern is cited by their carers too [7].

In general, most falls after stroke are reported during the daytime [2, 3], in the patient’s own room and in the bathroom/toilet area. In acute and inpatient rehabilitation, most falls are reported during transfers. In the community, falls occur most frequently during walking (predominantly indoors) [2]. Loss of balance, misjudgement and foot dragging during either walking, turning and sit to stand transfers were reported by fallers as the suspected causes and activities leading to falls [8].

Walking aids are often used to improve stability and walking following stroke [9, 10]. Ankle-foot orthoses (AFOs) are prescribed to provide mediolateral stability in stance, facilitate toe-clearance in swing and promote heel strike [11]. Positive effects of AFOs on mobility, walking speed and balance are reported [12, 13]. Furthermore, beneficial effects on knee [14] and ankle kinematics [14–16] are found. As a result, AFO-use may positively influence the prevalence of falls. However, literature studying the effects of AFOs in relation to falls after stroke is limited. AFO-use is reported to decrease the fear of falling [17, 18] and to improve balance confidence [19]. Cakar et al. reported that “AFOs seem to be a good supportive choice for the fall risk reduction in chronic stroke patients” [10]. Several balance and fall risk tests were performed with and without AFO in their study, but actual falls were not included as an outcome measure. Prospective intervention studies assessing effects of AFOs on falls are lacking. Three review studies on falls in patients after stroke concluded that there is limited evidence as to whether interventions for preventing falls (including studies assessing effects of AFOs) are effective and recommended further research [2, 3, 20].

This paper aims to study the effects of early AFO-provision on the occurrence and circumstances of falls and near falls in patients after stroke. The results from this current paper originate from a larger randomized controlled trial, in which specifically the effects of the timing of the provision of AFOs early or delayed after stroke (study week 1 vs week 9) were studied and several outcome levels were used. The effects of timing of providing AFOs on clinical scales [13, 21], lower limb kinematics [15, 16] and tibialis anterior muscle electromyography [22] have been previously reported. In the current paper, the early group (provided with AFOs after inclusion), was compared with the delayed group (not yet provided with AFOs) for eight weeks. Furthermore, follow-up measurements were performed in which occurrence and circumstances of falls/near falls were studied from week 9 to 52. In this period, both groups were provided with AFOs.

## Methods

Study data were collected as part of a single center, randomized, controlled, parallel group study. The study was approved by the Medical Ethical Committee Twente, registered in “the Netherlands Trial Register”, number NTR1930 and followed the CONSORT-guidelines [23]. All subjects provided written informed consent.

### Subjects

Subjects were recruited by the main researcher between December 2009 and March 2014, follow-up continued until 2015. We recruited subjects from the Roessingh, Center for Rehabilitation in Enschede, the Netherlands. Inclusion criteria were: 1) unilateral ischemic or hemorrhagic stroke leading to hemiparesis (single and first-ever stroke or history of previous stroke with full physical recovery); 2) indication for AFO-use (i.e. abnormal initial floor contact and/or problems with toe-clearance in swing and/or impaired ability to take bodyweight through the paretic lower limb in stance) determined by the treating rehabilitation physician and physiotherapist; 3) maximal six weeks post-stroke; 4) minimal 18 years; 5) receiving in-patient rehabilitation care at inclusion; 6) able to follow simple verbal instructions. Subjects suffering from severe comprehensive aphasia, neglect or cardiac, pulmonary or orthopedic disorders that could interfere with gait were excluded.

### Randomization

An independent person, not involved in the study, allocated participants to one of two intervention-groups using stratified block-randomization: 1) AFO-provision at inclusion, in study week 1 (early group); or 2) AFO-provision eight weeks later, in study week 9 (delayed group). Randomization was performed with sealed envelopes in blocks of four with ratio 1:1. Stratification was based on the Functional Ambulation Categories (FAC) [24]. Walking with (FAC 0–2) and without (FAC 3–5) physical support of another person at inclusion were used as stratification categories before randomization.

### AFO-provision

Subjects were provided with one of three commonly used types of off-the-shelf, non-articulated, posterior leaf design, polyethylene or polypropylene AFOs: flexible, semi-rigid or rigid (Basko Healthcare, Zaandam, the Netherlands). All orthoses were worn inside the shoe and included a proximal calf strap. AFO-fitting was performed by a licensed orthotist. The same orthotist was involved during the entire study. The type of AFO was chosen in week 1 (early group) or week 9 (delayed group) according to a custom developed protocol [13]. The effect of the prescribed AFO was verified and confirmed by the responsible physician in all subjects. After AFO-provision, subjects were instructed to use the AFO throughout the day, including during their stay at the ward, during therapies and when subjects went home. Besides the AFO-intervention, all subjects received usual care from experienced physiotherapists according to the Dutch guidelines for physiotherapy after stroke [25]. All subjects started with inpatient rehabilitation. If necessary, patients followed outpatient rehabilitation after discharge.

### Falls and near falls definitions

A fall was defined as “an event that results in a person coming to rest unintentionally on the ground or other lower level, not as a result of a major intrinsic event of overwhelming hazard” [26]. A near fall was defined as “an occasion on which an individual felt that they were going to fall but did not actually do so” [27]. Falls and near falls were excluded if they were not related to gait, for example a fall with a bicycle.

## Data-collection

At inclusion, basic demographic data were recorded. The number of falls/near falls during the first year after inclusion was assessed using a diary. The diary was filled in weekly during the first 17 weeks of the study. Subsequently, in week 26 the diary was filled in to cover study week 18–26, and in week 52, to cover week 27–52 of the study. A researcher or physiotherapist involved in the study, visited the subject to make sure that the diary was completed each time. In the diary, the number of falls, near falls and actual AFO-use were registered. For every reported incident, information related to the circumstances of the incident was obtained through open-ended questions to establish whether the AFO was used during the incident, the location of the incident, the activity being performed and possible injuries. This information was then further categorized: 1) AFO-use (with/without); 2) location (rehabilitation center/home-inside/outside/other); 3) activity (transfer/standing/walking); and 4) consequences (none/fear of falling/mild physical/severe physical), see Table 1. “Unknown” was noted when the diary was incomplete, for example when the subject remembered the fall, but did not remember whether the AFO was used.

**Table 1:** Categories used to determine fall and near fall conditions.

Main categories	AFO-condition	Location	Activity	Consequences
Sub-categories	- With AFO - Without AFO - Unknown	- Rehabilitation center - Home environment (inside) - Outside - Other - Unknown	- Transfers - Standing - Walking - Unknown	- No consequences - Fear of falling (such as becoming startled or anxious for new incidents) - Mild physical consequences (bruises or grazes) - Severe physical consequences, requiring medical care (such as fractures or cuts) - Unknown

As part of the longitudinal study, walking independence and balance were assessed using the Functional Ambulation Categories (FAC) [24] and the Berg Balance Scale (BBS) [28], respectively. FAC scores ≤2 indicate that a subject requires physical support, or verbal supervision (FAC 3) for functional ambulation. BBS scores below 45 points are related to increased fall-risk [29]. The FAC and BBS were assessed biweekly for the first 17 weeks, and a follow-up measurement was performed in week 26. All falls/near falls were labelled with the last known BBS and FAC score. For instance, a fall incident in week 6 was labelled with the score from week 5, an incident in week 18–25 with the score from week 17. All incidents in week 26–52 were labelled with the scores from week 26. The early group performed the tests without AFO in week 1, and in the other weeks with AFO. The delayed group performed the tests without AFO until week 9, and from week 11 with AFO.

### Statistical analysis

SPSS version 19 (IBM SPSS Statistics, Chicago, IL, USA) was used for data-analysis. The level of significance for all analyses was set at *p*<0.05. No power-calculation was performed, since relevant data regarding timing of AFO-provision were not available.

Basic demographic data of the early and delayed groups at inclusion were compared using independent samples t-test/Mann-Whitney U test for continuous variables and chi-squares test/Fisher exact test for categorical variables, as appropriate. The person-time fall/near fall incidence rate (number of falls/near falls per person week, per study group, per period) was calculated by dividing the total number of falls or near falls, by the sum of the total amount of measurement weeks. This was calculated for the early and delayed group separately, for study week 1–8 and study week 9–52. To answer the research question, occurrence of number of falls and near falls in study week 1–8 were compared between the early (who had already been provided with AFOs) and delayed group (who had not yet been provided with AFOs). Furthermore, the occurrence of falls and near falls in study week 9–52 were compared. In this period, both groups of subjects had been provided with AFOs. Poisson regressions were chosen in case of mean-variance equality, negative binomial regressions (generalized Poisson regression) were used for over-dispersed count data (variance exceeded mean number of incidents). Because subjects reported relatively low number of incidents, fall/near fall circumstances and related FAC and BBS scores were only presented in a descriptive way. All obtained data were included in the analysis. Therefore, a participant was included in the number of patients if at least one week in the corresponding period was filled in.

## Results

### Baseline

**Fig 1:**
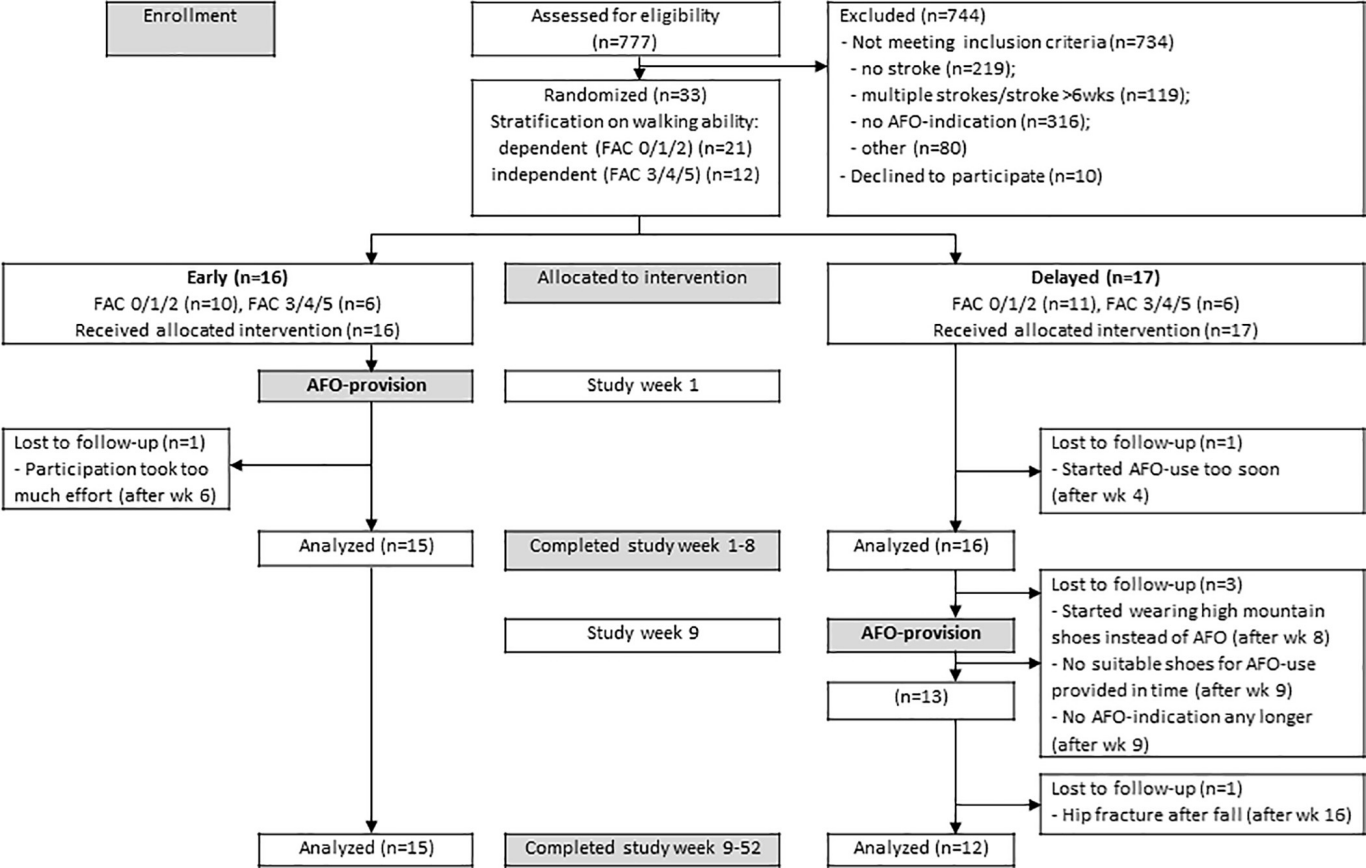
CONSORT-flowchart. The figure shows the participant flow through the study. Abbreviations: AFO: ankle-foot orthoses; FAC: Functional Ambulation Categories.

In total 33 subjects were included in the study, 16 in the early group and 17 in the delayed group. No statistically significant differences were found between both groups at baseline (see Table 2). Fig 1 details the participants flow through the study. Before completing the first eight weeks, in which the early group had been provided with an AFO and the delayed not yet, two subjects (one early, one delayed) dropped out. After week 8, one subject (delayed) dropped out. Of the 30 subjects (15 early, 15 delayed) that were included in the period week 9–52, three subjects (in the delayed group) dropped out. Two of them dropped out already after week 9, at the moment of AFO-provision. Of the thirteen subjects in the delayed group that had been provided with an AFO, one dropped out after week 16. In total, 27 subjects (15 early and 12 delayed) completed the study until week 52. Six weeks of diaries entries, involving four subjects (two early, two delayed), are missing due to illness or vacation.

**Table 2:** Subject characteristics at inclusion.

		Total (n = 33)	Early (n = 16)	Delayed (n = 17)
Sex (male/female)^a^		20 / 13	10 / 6	10 / 7
Age (years)^b^		57.2 (9.2)	56.9 (9.6)	57.5 (9.1)
Time since stroke at inclusion (days)^b^		31.4 (6.3)	32.0 (6.2)	30.8 (6.5)
FAC-level at inclusion (0/1/2/3/4/5)^c^		0 / 7 / 14 / 11 / 1 / 0	0 / 3 / 7 / 6 / 0 / 0	0 / 4 / 7 / 5 / 1 / 0
Affected body side (left/right)^a^		16 / 17	8 / 8	8 / 9
Type of stroke (ischemic/haemorrhagic)^c^		27 / 6	14 / 2	13 / 4
Type of AFO at provision* (flexible/semi-rigid/rigid/no orthosis)^c^		27 / 0 / 3 / 3	14 / 0 / 2 / 0	13 / 0 / 1 / 3
Sensation**	Tactile (normal/impaired/absent)^c^	26 / 4 / 3	13 / 1 / 2	13 / 3 / 1
	Propriosepsis (normal/impaired/absent)^c^	26 / 6 / 1	13 / 2 / 1	13 / 4 / 0
Mini-Mental State Examination^d^		27.0 (23.5–28.0)	27.0 (25.3–28.0)	27.0 (22.5–28.0)

Abbreviations: FAC: Functional Ambulation Categories; AFO: ankle-foot orthosis. Mean (SD) or median (interquartile range) are presented.

^a^ Pearson chi-squared test (2-tailed)

^b^ independent samples t-test

^c^ Fisher exact test

^d^ Mann-Whitney U test with median (IQR)

* Three subjects were not provided with an orthosis: one dropped-out before orthosis-provision (after week 4); one preferred wearing high mountain shoes instead of an orthosis and one had no longer an indication for an AFO at the moment of provision in week 9. Furthermore, two subjects (both early) changed from a flexible to a semi-rigid type during the study (in week 4 and 8, respectively) since support provided by the flexible type appeared to be insufficient.

**tested with Erasmus MC modifications to the Nottingham Sensory Assessment, lower limb part.

### AFO-compliance

In general, subjects reported that they used the AFO from the week of prescription, i.e. study week 1 or 9 for the early and delayed group, respectively. One subject (delayed) experienced pressure sores and did not wear the AFO for five weeks (week 10–11 and 13–15). Three participants stopped using the AFO in the period of week 18–26 (two early, one delayed). These subjects reported that they felt that they did not need the AFO anymore because their walking pattern improved. In the period of week 27–52 five subjects stopped using the AFO (one early, four delayed). Four because they felt they did not need the AFO anymore, two of them (both delayed) preferred to use a cane instead of an AFO, and orthopedic shoes instead of an AFO were provided.

### Study week 1–8

#### Falls and near falls

Nine out of 16 subjects in the early group (56.3%), compared to four out of 17 subjects in the delayed group (23.5%) experienced at least one fall during the first eight weeks of the study, see Table 3. Nine subjects in the early group reported 11 falls during this period, of which one subject reported three falls. Four subjects in the delayed group (who had not use an AFO yet) reported each one fall. Out of the 16 subjects in the early group, five (31.1%) reported a near fall, one each. Six out of 17 subjects in the delayed group (35.5%) reported in total seven near falls. Person-time fall incidence rate was 0.087 and 0.031 per person week, for the early and delayed group, respectively. For near falls the person-time incidence rate was 0.040 and 0.054, for the early and delayed group, respectively. Falls occurred significantly more in the early group compared to the delayed group, with an Incidence Rate (IR) of 0.69 for the early, and 0.24 for the delayed group (Incidence Rate Ratio (IRR) = 2.92; 95%CI = 1.06–8.08, *p* = 0.039). No significant differences were found in near fall rates (IR 0.31 (early) and 0.41 (delayed) (IRR = 0.76; 95%CI = 0.28–2.07, *p* = 0.59).

**Table 3:** Total number of falls and near falls in week 1–8 and 9–52.

		Week 1–8				Week 9–52			
Falls		Near falls		Falls		Near falls	
		Early	Delayed	Early	Delayed	Early	Delayed	Early	Delayed
Number of subjects	At start interval	16	17	16	17	15	15*	15	15
	Completed interval	15	16	15	16	15	12	15	12
Number of incidents reported		11	4	5	7	12	14	16	9
Number of subjects reporting incidents (%)		9 (56.3)	4 (23.5)	5 (31.3)	6 (35.3)	8 (53.3)	10 (66.7)	8 (53.3)	5 (33.3)
Median number of incidents per subject (min-max)		1.0 (0–3)	0.0 (0–1)	0.0 (0–1)	0.0 (0–2)	1.0 (0–3)	1.0 (0–4)	1.0 (0–4)	0.0 (0–4)
Total number of measurement weeks		126	130	126	130		656	538	656	538
Person-time incidence rate (number of incidents per person week, per study group, per period		0.087	0.031	0.040	0.054	0.018	0.026	0.024		0.017

*two subjects dropped out after week 9 and were included for only one week in this interval.

#### Fall and near fall circumstances

Despite the fact that AFOs were provided in week 1–8 in the early group, seven out of 11 falls (63.6%) and two out of five near falls (40%) in the early group occurred without wearing the AFO, see Table 4. Conforming to the protocol, all falls and near falls reported in the delayed group were without using an AFO. No remarkable differences were found in locations of the incidents between the early and delayed group, all falls/near falls occurred in the rehabilitation centre or inside the home. The majority of the falls occurred during transfers or standing (early 36.4% transfers, 27.3% standing; delayed 75.0% transfers). Only one fall (9.1% early; 25.0% delayed) and near fall (20.0% early; 14.3% delayed) in each group were related to walking. Most falls (72.7% early; 50.0% delayed) and near falls (100.0% early; 85.7% delayed) in week 1–8 did not have serious consequences.

**Table 4:** AFO-conditions, locations, activities and consequences related to falls and near falls in week 1–8 and 9–52.

		Week 1–8								Week 9–52							
		Falls				Near Falls				Falls				Near Falls			
		Early		Delayed		Early		Delayed		Early		Delayed		Early		Delayed	
**Number of subjects**	at start interval^a^	16		17		16		17		15		15		15		15	
	completed interval	15		16		15		16		15		12		15		12	
**Number of incidents**		11		4		5		7		12		14		16		9	
		NoI	%	NoI	%	NoI	%	NoI	%	NoI	%	NoI	%	NoI	%	NoI	%
**AFO-use**	With AFO	3	27.3	n.a.		3	60.0	n.a.		3	25.0	5	35.7	11	68.8	4	44.4
	Without AFO	7	63.6	4	100.0	2	40.0	7	100.0	8^b^	66.7	9^c^	64.3	4^d^	25.0	3	33.3
	Unknown	1	9.1	n.a.		-	-	n.a.		1	8.3	-	-	1	6.3	2	22.2
	Total		100.0		100.0		100.0		100.0		100.0		100.0		100.0		100.0
**Locations**	Rehab. center	6	54.5	2	50.0	4	80.0	5	71.4	2	16.7	2	14.3	1	6.3	3	33.3
	Home (inside)	5	45.5	1	25.0	1	20.0	1	14.3	8	66.7	8	57.1	9	56.3	4	44.4
	Outside	-		-	-	-	-	-	-	1	8.3	3	21.4	5	31.3	1	11.1
	Other	-		-	-	-	-	-	-	-	-	1^e^	7.1	-	-	1^e^	11.1
	Unknown	-		1	25.0	-	-	1	14.3	1	8.3	-	-	1	6.3	-	-
	Total		100.0		100.0		100.0		100.0		100.0		100.0		100.0		100.0
**Activities**	Transfers	4	36.4	3	75.0	1	20.0	3	42.9	1	8.3	2	14.3	-	-	5	55.6
	Standing	3	27.3	-	-	2	40.0	2	28.6	4	33.3	5	35.7	3	18.8	1	11.1
	Walking	1	9.1	1	25.0	1	20.0	1	14.3	5	41.7	6	42.9	9	56.3	3	33.3
	Unknown	3	27.3	-	-	1	20.0	1	14.3	2	16.7	1	7.1	4	25.0	-	-
	Total		100.0		100.0		100.0		100.0		100.0		100.0		100.0		100.0
**Consequences**	None	8	72.7	2	50.0	5	100.0	6	85.7	1	8.3	7	50.0	11	68.8	6	66.7
	Fear of falling	1	9.1	1	25.0	-	-	-	-	-	-	2	14.3	3	18.8	2	22.2
	Mild physical	2	18.2	1	25.0	-	-	1	14.3	5	41.7	4	28.6	2	12.5	1	11.1
	Severe physical	-	-	-	-	-	-	-	-	5	41.7	1	7.1	-	-	-	-
	Unknown	-	-	-	-	-	-	-	-	1	8.3	-	-	-	-	-	-
	Total		100.0		100.0		100.0		100.0		100.0		100.0		100.0		100.0

Abbreviations: NoI, number of incidents per AFO-condition, location, activity or consequences; %, percentage of incidents per AFO-condition, location, activity, or consequences; n.a., not applicable, since the delayed group was not provided with an AFO in this period.

^a^ A participant is included in the number of participants if at least one week of the diary is completed during the corresponding period.

^b^ One of these falls was experienced by a participant who had already stopped wearing the AFO.

^c^ Two of these falls were experienced by one participant who already stopped wearing the AFO.

^d^ Two of these near falls were experienced by two participants who already stopped wearing the AFO.

^e^ Locations were categorized as “other” when incidents took place at locations other than the rehabilitation center, at home (inside) or outside. In this case at the library and at work.

#### Walking independence and balance levels

**Fig 2:**
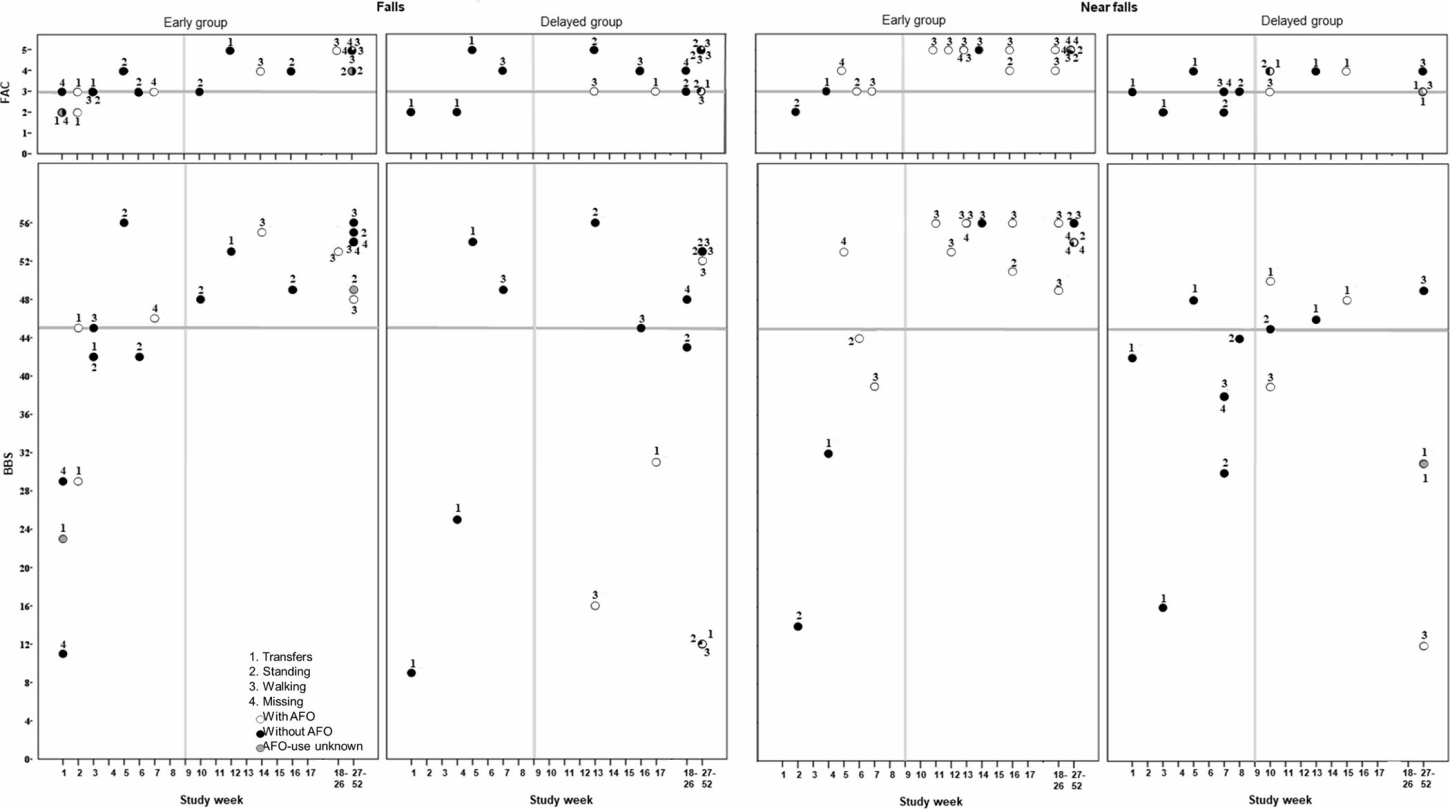
Functional Ambulation Categories (FAC) and Berg Balance Scale (BBS) scores related to falls and near falls in the early and delayed group. The position of the circle represents the BBS- or FAC-score at the time of the incident. The colour of the circle represents whether the AFO was used at the time of the incident. The numbers around the circles represent the activities performed during the incident. The vertical grey lines represent study week 9 (before week 9 the delayed group had not use an AFO), the horizontal grey lines indicate FAC-scores of 3 and BBS-scores of 45.

In week 1–8, 10 out of the 11 falls (90.9%) in the early group occurred when participants had a FAC-score ≤3, see Fig 2. This score indicates that a subject required physical support or verbal supervision for functional ambulation. In six of these incidents, the AFO was not used. Incidents were reported during transfers (n = 4), standing (n = 2) and walking (n = 1). In the delayed group two out of the four falls (both during transfers) were related to a FAC-score ≤3. BBS-score was <45 in seven out of the 11 falls in the early group in week 1–8, compared to two out of the four falls in the delayed group. AFOs were not used in respectively five and two of these falls.

### Study week 9–52

#### Falls and near falls

During week 9–52, when both groups were provided with an AFO, eight of the 15 subjects in the early group (53.3%) reported in total 12 falls (1–2 falls per person), see Table 3. The delayed group reported 14 falls, which were experienced by 10 of the 15 subjects (66.7%), (1–3 falls per person). Near falls were reported by eight of the 15 subjects in the early group (53.3%) and by five of the 15 subjects in the delayed group (33.3%). In total, 16 and nine near falls were reported by the early and delayed group, respectively, 1–4 near falls per person. In the early and delayed group, the person-time fall incidence rate was 0.018 and 0.026 falls per person week, respectively. The person-time incidence rate for near falls in the early and delayed group was 0.024 and 0.017, respectively. Statistical analysis did not show any significant differences between the groups with respect to falls (IR 0.80 (early) and 0.92 (delayed) (IRR = 0.86; 95% CI = 0.39–1.89, *p* = 0.702)) and near falls (IR 1.07 (early) and 0.60 (delayed) (IRR = 1.79; 95%CI = 0.59–5.39, *p* = 0.310)).

#### Fall and near fall circumstances

No significant differences were established in fall and near fall circumstances between both groups in week 9–52, see Table 4. In this period all subjects had been provided with an AFO. However, approximately two-third of the falls occurred while the AFO was not used (66.7% early; 64.3% delayed). Near falls were mainly reported whilst using the AFO (68.8% early; 44.4% delayed). Both groups reported that most falls/near falls took place inside the home (falls 66.7% early and 57.1% delayed; near falls 56.3% early and 44.4% delayed). One (8.3%) and three (21.4%) falls in the early and delayed group, and five (31.3%) and one (11.1%) near falls were reported outside the home in week 9–52, whereas in week 1–8 no incidents were reported outside. The highest number of fall incidents in both the early and delayed group occurred whilst walking (41.7% early; 42.9% delayed), and approximately one-third of the incidences took place while standing. Near falls occurred primarily during walking (56.3%) in the early group. In the delayed group this was during transfers (55.6%). Noteworthy is that in contrast to week 1–8, a total of six falls resulted in severe injuries (five early (41.7%); one delayed (7.1%)). Three fractures were reported (two early (ankle fracture, without AFO; humerus fracture, with AFO); one delayed (hip fracture, without AFO)). Furthermore, a painful foot required an X-ray, a painful shoulder required a medical check and a cut in the face required stitches. Most near falls were reported without consequences.

#### Walking independence and balance levels

In week 9–52, one out of the 12 falls in the early group occurred with FAC-score ≤3, see Fig 2. For the delayed group this was six out of the 14 falls. The majority of these falls were without AFO. BBS-score was ≥45 in all 12 falls in the early group, while six out of 14 were <45 in the delayed group.

## Discussion

The current paper studied the effects of AFO-provision on the occurrence and circumstances of falls and near falls after stroke. We found that during the first eight weeks of the study, falls occurred significantly more often in patients who had been provided with AFOs, compared to patients who had not yet been provided with AFOs. However, it is important to note that in 63.6% of the falls in patients who had been provided with an AFO, the AFO was not worn at the time of the incident. In week 9 to 52, when all subjects had been provided with an AFO, there was no difference in the number of falls between the groups. No effects on near falls were found between the groups, both in week 1–8 and week 9–52. Although the actual number of falls were relatively low, serious injuries including fractures were reported.

To the best of our knowledge, this is the first prospective study on the effects of AFO-provision on falls early after stroke. We established that 13 subjects (nine early, four delayed) experienced at least one fall in week 1–8 of our study, which is 39.3%. In week 9–52 this was 60.0% (18 subjects in total; eight early, 10 delayed). These numbers are in accordance with previous studies on falls after stroke, reported rates of patients falling during inpatient rehabilitation from 10.5%-47.0% [2]. Rates of 37%-73% and 43%-70% during the first six months after discharge from hospital [3] and 1-year follow-up [2] are reported, respectively. We found near fall rates of 33.3% for the subjects in week 1–8 and 48.1% in week 9–52. Interestingly, our findings are less than the almost 80% previously reported by Hyndman et al. [8]. However, they included subjects living at home for at least 3 months following the stroke and results were obtained by interviews including frequency and circumstances of falls/near falls from the past 12 months.

The current study found that falls occurred more frequently in subjects who had been provided with AFOs early after stroke. Based on these results, one may be inclined to conclude that it is not beneficial to use AFOs early after stroke. However, further analysis of this information is needed. The clinical reasoning used in the consideration for the provision of AFOs early after stroke is multifaceted and therefore it would be remiss not to examine the other aspects and potential merits. We previously reported the beneficial effects of AFOs on other outcome levels. Principally, we established that early AFO-provision tends to results in higher functional levels (i.e. higher levels of independence of walking and higher balance levels) earlier on in the rehabilitation [21]. This can be important in order to perform task-specific rehabilitation exercises with high repetitions early in the rehabilitation, which is known to be important for improved outcomes [25]. These beneficial effects on functional outcomes were found without negative consequences of AFO-use on muscle activity of the tibialis anterior [22]. In addition, decreased foot-clearance is reported as an increased risk for stumbling and falls [2]. AFOs are provided to improve foot-clearance in swing phase [11], under the assumption that this improves safety of walking. It is possible that the AFOs provided did not improve foot-clearance sufficiently. However, we previously reported on kinematic effects of AFOs on the gait pattern [15, 16]. These studies included the same subjects as the current study, and confirmed that the ankle was sufficiently corrected. Therefore, insufficient correction of foot-clearance during walking because of AFO-use cannot explain the higher number of falls that were reported in the first eight weeks of our study by the group that was provided with AFOs.

We suggest that the higher number of falls we established in the early group after been provided with AFOs can most likely be explained by factors related to the situations in which falls occurred and to patient characteristics, rather than to the AFO. We advise that these factors be taken into consideration when considering the benefits of using or not using AFOs early after stroke. Importantly, in seven out of 11 falls (63.6%) the AFO was not used, despite the fact that the subjects reported that they used the AFO when diaries where completed. A closer analysis of the data revealed that most falls without AFO (five out of seven) occurred in the bed- and bathroom, during activities related to getting dressed, getting out of bed during the night, toileting and showering. These are all situations which are often related to walking on bare feet, and in which it may not be possible to wear AFOs (such as showering), or at least would be very inconvenient to use an AFO (for example transfer out of bed at night). As a results, falls without AFO happen, even when AFOs are used as they should be. Secondly, the majority of the falls (90.9%) in week 1–8 were reported while the related level of walking was a FAC-score 3 or lower, in combination with lower levels of balance (often <45 on BBS), see Fig 2. This implies that at least verbal supervision or stand-by help without physical contact from another person should have been provided to these subjects at the time of the fall. Nyberg et al. previously reported that in 58% of the falls after stroke at a stroke rehabilitation unit, patients acted against instructions by the rehabilitation team (such as transferring or walking without recommended supervision or aids used) [30]. Ignoring instructions by patients, or unclear instructions by clinicians, could very likely explain the high number of falls related to low FAC-levels in our population. In addition, we previously reported trends of higher levels of independence of walking and higher balance levels earlier on in the rehabilitation [21]. As a result, the early group is hypothetically also more likely to fall, as they are more active earlier on in the rehabilitation. Attention deficits (as part of cognitive deficits) are common after stroke and are correlated to falls [31, 32]. Although this was not part of our study and therefore speculative, the higher activity-levels earlier on in the rehabilitation, in combination with possible attention deficits often present early after stroke, are probably important aspects to take into consideration in the case AFOs are provided early after stroke. Perhaps some patients are physically able to perform mobility-tasks independently due to early AFO-provision, but their cognitive function may not yet be sufficient. Based on our results, we would like to emphasize the importance of careful instructions from clinicians and nursing staff to patients and their relatives, and to emphasize the potential risks of performing activities without the proper assistance, especially in situations in which an AFO is not worn.

Another important finding in our study is that, although AFOs are often provided to improve safety during walking, the majority of fall incidents between week 1–8 occurred during transfers and standing. This is in accordance with previous literature on falls after stroke [30]. The main mechanisms to prevent falls in these situations, are thought to be accurate balance reactions (including the ability to use arms and hands to grasp for example a (wheel)chair) and AFOs are not likely to contribute to this. This finding highlights the importance, as previously mentioned, of careful instructions. In daily clinical practice, the decision on whether patients are able to ambulate independently is often made based on walking ability whilst wearing an AFO. Based on our results, we would like to emphasize the importance of targeting safe transfers and standing during therapy, even when subjects have already reached independent walking levels. Furthermore, practicing daily life activities on bare feet should be taken into consideration. Again, careful instructions by clinicians and nursing staff to patients and their family are important in these activities. This includes the effects and differences of performing activities with or without AFO, taking the cognitive level of the patient into consideration. All these factors may be subject of future research aiming to reduce fall incidence after stroke.

An important strength of our study is that we prospectively included the provision of AFOs in relation to falls after stroke. To the best of our knowledge, this is the first time. Previous studies on falls after stroke did not include the effects of AFO-use [2, 3, 20]. Only one study on the effects of AFOs included outcome measures related to falls [10]. However, actual falls were not included as outcome measure in this study. We prospectively assessed falls and near falls on a weekly basis during the first 17 weeks of the study, thereby limiting recall bias as much as possible in this period.

A major study-limitation is the small sample size. Within this limited sample size, the actual number of falls and near falls reported were relatively low. Therefore, no statistical analysis could be performed in relation to the falls/near fall circumstances and related FAC- and BBS-scores. The power was sufficient for falls in study week 1–8, significant results were found. Outcomes for near falls in week 1–8, and falls and near falls in week 9–52 were found non-significant. Post-hoc power analysis with the reported hazard ratios (IRR 0.76, IRR 0.86 and IRR 1.79, respectively) resulted in a power of 8% for near falls in week 1–8, and 16% and 14% for falls and near falls in week 9–52, respectively, confirming that the sample size was not sufficient for these outcome parameters. We included participants in our analysis in case at least one week was completed in the diary in a particular period. Two subjects (both delayed) dropped-out after week 9 of the study. Thus they only contributed one week of data to the period of week 9–52. Post-hoc analysis without these two subjects showed that conclusions with respect to IRR for falls and near falls in week 9–52 did not change. Despite the short recall period of one week during a large part of the study, there were some situations in which the subjects could not remember the exact circumstances of the fall/near fall. In addition, diaries of week 26 and 52 included a large recall period, from week 18–26 and week 27–52, respectively. This may have affected the results. Furthermore, results with respect to BBS- and FAC-scores in relation to falls in week 26–52 must be interpreted with caution, as incidents reported in this period were labelled with BBS- and FAC-scores from week 26. BBS and FAC were not recorded after week 26. Therefore, potential changes in BBS and FAC after week 26 were not registered. In addition, some subjects in our study stopped using the AFO because they felt it was no longer required. This was documented previously as an important reason to stop using AFOs [33]. However, falls and near falls reported by these subjects after stopping using their AFO were included in our analysis, which may have affected our results. Finally, it was not possible to blind subjects and assessor for AFO-use.

## Conclusions

This study showed that falls occurred significantly more often in patients who had been provided with AFOs early after stroke, compared to subjects who had not yet been provided with AFOs. However, it is important to notice that in the subjects who had been provided with AFOs, 63.6% of the falls in the first eight weeks of the study occurred without wearing the AFO. The majority of these incidents took place during standing and transfers, while subjects had no independent walking ability and had low levels of balance. Beneficial effects of early AFO-provision on clinical scales and ankle-kinematics were reported previously. The current study highlights the importance of careful instructions by clinicians and nursing staff to patients and their relatives concerning AFO-use early after stroke, and which activities can, and maybe even more importantly, cannot be performed independently. Whether the AFO is used or not in these activities, is of considerable importance when instructing patients.

## Supporting information

S1 Appendix Data. (XLSX)

S2 Appendix Previous publications. (PDF)

S3 Appendix CONSORT checklist. (PDF)

S4 Appendix Study protocol. (PDF)
