# Supplementary material for: The effect of ankle-foot orthoses on fall/near fall incidence in patients with (sub-)acute stroke: A randomized controlled trial
Source: PLoS One. 2019 Mar 12;14(3):e0213538. doi: 10.1371/journal.pone.0213538 (PMC6414023; doi:10.1371/journal.pone.0213538)
Supplement: S2 Appendix — (PDF) [file pone.0213538.s002.pdf]

Supporting information file

Previously published articles within the randomized controlled trial

Early or delayed provision of an ankle-foot orthosis in patients with acute and subacute stroke: A randomized controlled trial

Corien DM Nikamp, Jaap H Buurke, Job van der Palen, Hermie J Hermens and Johan S Rietman

Clinical Rehabilitation 2017, Vol. 31(6) 798-808
DOI: 10.1177/0269215516658337
Received: 17 November 2015; accepted: 10 June 2016

Abstract
Objective: (1) To study the effects of providing ankle-foot orthoses in subjects with (sub)acute stroke; and (2) to study whether the point in time at which an ankle-foot orthosis is provided post-stroke (early or delayed), influences these effects.
Design: Randomized controlled trial.
Setting: Rehabilitation centre.
Subjects: Unilateral hemiparetic stroke subjects with indication for use of an ankle-foot orthosis and maximal six weeks post-stroke.
Interventions: Subjects were randomly assigned to: early provision (at inclusion; week 1) or delayed provision (eight weeks later; week 9).
Outcome measures: 10-metre walk test, 6-minute walk test, Timed Up and Go Test, stairs test, Functional Ambulation Categories, Berg Balance Scale, Rivermead Mobility Index and Barthel Index; assessed in week 1, 3, 9 and 11.
Results: A total of 33 subjects were randomized (16 early, 17 delayed). Positive effects of ankle-foot orthoses were found two weeks after provision, both when provided early (significant effects on all outcomes) or delayed (Berg Balance Scale p=0.011, Functional Ambulation Categories p=0.008, 6-minute walk test p=0.005, Timed Up and Go Test p=0.028*)*. Comparing effects after early and delayed provision showed that early provision resulted in increased levels of improvement on Berg Balance Scale (+5.1 points, p=0.002), Barthel Index (+1.9 points, p=0.002) and non-significant improvements on 10-Meter walk test (+0.14m/s, p=0.093) and Timed Up and Go Test (-5.4 sec, p=0.087), compared to delayed provision.
Conclusions: We found positive effects of providing AFOs in (sub)acute stroke subjects that had not used these orthoses before.

Keywords: Ankle-foot orthosis, stroke rehabilitation, functional outcome, timing of provision, randomized controlled trial

Six-month effects of early or delayed provision of an ankle-foot orthosis in patients with (sub-)acute stroke: A randomized controlled trial

Corien DM Nikamp, Jaap H Buurke, Job van der Palen, Hermie J Hermens, Johan S Rietman

Clinical Rehabilitation 2017, Vol. 31(12) 1616-1624
DOI: 10.1177/0269215517709052
Received: 21 March 2017; accepted: 14 April 2017

Abstract

Objective: 1) Study the six-month clinical effects of providing ankle-foot orthoses at different moments (early or delayed) in (sub-)acute stroke; this is a follow-up to a published trial.
Design: Randomized controlled trial.
Setting: Rehabilitation centre.
Subjects: Unilateral hemiparetic stroke subjects maximal six weeks post-stroke with indication for ankle-foot orthosis use.
Interventions: Subjects were randomly assigned to early (at inclusion; week 1) or delayed provision (eight weeks later; week 9).
Outcome measures: Functional tests assessing balance and mobility were performed bi-weekly for 17 weeks and at 26 weeks.
Results: In all, 33 subjects were randomized. No differences at week 26 were found between both groups for any of the outcome measures. However, results suggest that early provision leads to better outcomes in the first 11-13 weeks. Berg Balance Scale (*p*=0.006), Functional Ambulation Categories (*p*=0.033) and 6-Minute Walk Test ($p<0.001$) showed significantly different patterns over time. Clinically relevant but non-significant differences of 4-10 weeks in reaching independent walking with higher balance levels were found, favouring early provision.
Conclusions: No six-month differences on functional outcomes of providing AFOs at different moments in the early rehabilitation after stroke were found. Results suggest that there is a period of 11-13 weeks in which early provision may be beneficial, possibly resulting in earlier independent and safe walking. However, our study was underpowered, therefore further research including larger numbers of subjects is warranted.

Keywords: Ankle-foot orthosis, stroke, six-month effect, timing of provision, randomized controlled trial

A randomized controlled trial on providing ankle-foot orthoses in patients with (sub-)acute stroke: Short-term kinematic and spatiotemporal effects and effects of timing

Corien DM Nikamp, Marte SH Hobbelink, Job van der Palen, Hermie J Hermens, Johan S Rietman, Jaap H Buurke

Gait & Posture 55 (2017) 15-22
https://doi.org/10.1016/j.gaitpost.2017.03.028
Received: 24 November 2016; received in revised form: 8 March 2017; accepted: 24 March 2017

Abstract
Initial walking function is often limited after stroke, and regaining walking ability is an important goal in rehabilitation. Various compensatory movement strategies to ensure sufficient foot-clearance are reported. Ankle-foot orthoses (AFOs) are often prescribed to improve foot-clearance and may influence these strategies. However, research studying effects of actual AFO-provision early after stroke is limited. We conducted an explorative randomized controlled trial and aimed to study the short-term effects of AFO-provision on kinematic and spatiotemporal parameters in patients early after stroke. In addition, we studied whether timing of AFO-provision influenced these effects. Unilateral hemiparetic patients maximal six weeks post-stroke were randomly assigned to AFO-provision: early (at inclusion) or delayed (eight weeks later). Three-dimensional gait-analysis with and without AFO in randomized order was performed within two weeks after AFO-provision. Twenty subjects (8 early, 12 delayed) were analyzed. We found significant positive effects of AFO-provision for ankle dorsiflexion at initial contact, foot-off and during swing (-3.6° (7.3) vs 3.0° (3.9); 0.0° (7.4) vs 5.2° (3.7); and -6.1° (7.8) vs 2.6° (3.5), respectively), all $p<0.001$. No changes in knee, hip and pelvis angles were found after AFO-provision, except for knee (+2.3°) and hip flexion (+1.6°) at initial contact, $p \leq 0.001$. Significant effects of AFO-provision were found for cadence (+2.1 steps/min, $p=0.026$), stride duration (-0.08 sec, $p=0.015$) and single support duration (+1.0%, $p=0.002$). Early or delayed AFO-provision after stroke did not affect results. In conclusion, positive short-term effects of AFO-provision were found on ankle kinematics early after stroke. Timing of AFO-provision did not influence the results.

Keywords: Ankle-foot orthosis, stroke rehabilitation, gait analysis, timing of provision, randomized controlled trial

The influence of early or delayed provision of ankle-foot orthoses on pelvis, hip and knee kinematics in patients with sub-acute stroke: A randomized controlled trial

Corien DM Nikamp, Job van der Palen, Hermie J Hermens; Johan S Rietman; Jaap H Buurke

Gait & Posture 63 (2018) 206-267
https://doi.org/10.1016/j.gaitpost.2018.05.012
Received: 3 August 2017; received in revised form: 3 May 2018; accepted: 9 May 2018

Abstract
Background: Compensatory pelvis, hip- and knee movements are reported after stroke to overcome insufficient foot-clearance. Ankle-foot orthoses (AFOs) are often used to improve foot-clearance, but the optimal timing of AFO-provision post-stroke is unknown. Early AFO-provision to prevent foot-drop might decrease the development of compensatory movements, but it is unknown whether timing of AFO-provision affects post-stroke kinematics.
Research questions: 1) To compare the effect of AFO-provision at two different points in time (early versus delayed) on frontal pelvis and hip, and sagittal hip and knee kinematics in patients with sub-acute stroke. Effects were assessed after 26 weeks; 2) To study whether possible changes in kinematics or walking speed during the 26-weeks follow-up period differed between both groups.
Method: An explorative randomized controlled trial was performed, including unilateral hemiparetic patients maximal six weeks post-stroke with indication for AFO-use. Subjects were randomly assigned to AFO-provision early (at inclusion) or delayed (eight weeks later). 3D gait-analysis with and without AFO was performed in randomized order. Measurements were performed in study-week 1, 9, 17 and 26.
Results: Twenty-six subjects (15 early, 11 delayed) were analyzed. After 26 weeks, no differences in kinematics were found between both groups for any of the joint angles, both for the without and with AFO-condition. Changes in kinematics during the 26-weeks follow-up period did not differ between both groups for any of the joint angles during walking without AFO. Significant differences in changes in walking speed during the 26-weeks follow-up were found (*p*=0.034), corresponding to the first eight weeks after AFO-provision.
Significance: Results indicate that early or delayed AFO-use post-stroke does not influence pelvis, hip and knee movements after 26 weeks, despite that AFO-use properly corrected drop-foot. AFOs should be provided to improve drop-foot post-stroke, but not with the intention to influence development of compensatory patterns around pelvis and hip.

Keywords: Ankle-foot orthosis, stroke rehabilitation, gait analysis, pelvis, hip and knee kinematics; compensatory movement patterns, randomized controlled trial

Effect of long-term use of ankle-foot orthoses on tibialis anterior muscle electromyography in patients with sub-acute stroke: A randomized controlled trial

Corien Nikamp, Jaap Buurke, Leendert Schaake, Job van der Palen, Johan Rietman, Hermie Hermens

Journal of Rehabilitation Medicine 2019; 51: 11-17
DOI: 10.2340/16501977-2498
Accepted: Sep 22, 2018; Epub ahead of print Oct 26, 2018

Abstract
Objective: To determine: 1) whether the use of ankle-foot orthoses over a period of 26 weeks affects tibialis anterior muscle activity; 2) whether the timing of provision of ankle-foot orthoses (early or delayed) affects results; 3) whether the provision of ankle-foot orthoses affects tibialis anterior-activity within a single measurement.
Design: Randomized controlled trial.
Subjects: Unilateral hemiparetic subjects, a maximum of six weeks post-stroke.
Methods: Subjects were assigned randomly to early (at inclusion; week 1) or delayed provision of ankle-foot orthoses (eight weeks later; week 9). Tibialis anterior electromyography was measured with and without ankle-foot orthoses, in study week 1, 9, 17 and 26.
Results: A total of 26 subjects were analyzed. In a single measurement, use of an ankle-foot orthosis significantly reduced the activity levels of the tibialis anterior muscle during the swing phase (p=0.041) compared with walking without an ankle-foot orthosis. During the 26-week follow-up, no changes were found in tibialis anterior muscle activity in the swing phase without an ankle-foot orthosis, both within-groups (p=0.420 early; p=0.282 delayed), and between-groups (p=0.987). After 26 weeks, no differences were found in tibialis anterior muscle activity between both groups in the swing phase, with (p=0.207) or without ankle-foot orthoses (p=0.310).
Conclusions: Use of ankle-foot orthoses post-stroke reduced tibialis anterior muscle activity in the swing phase within one measurement; however, long-term use of ankle-foot orthoses for 26 weeks did not affect such activity. Early or delayed provision of ankle-foot orthoses did not affect the findings. The results indicate that there is no need to fear negative consequences on tibialis anterior muscle activity because of long-term ankle-foot orthoses use (early) after stroke.

Keywords: Ankle-foot orthosis, stroke rehabilitation, muscle electromyography, tibialis anterior, long-term effects, timing of provision, randomized controlled trial
