# Supplementary material for: The effect of ankle-foot orthoses on fall/near fall incidence in patients with (sub-)acute stroke: A randomized controlled trial
Source: PLoS One. 2019 Mar 12;14(3):e0213538. doi: 10.1371/journal.pone.0213538 (PMC6414023; doi:10.1371/journal.pone.0213538)
Supplement: S4 Appendix — (PDF) [file pone.0213538.s004.pdf]

RESEARCH PROTOCOL

Timing of providing ankle-foot orthoses in stroke rehabilitation

PROTOCOL TITLE 'Timing of providing ankle-foot orthoses in stroke rehabilitation'

Protocol ID	NL28347.044.09
Short title	Timing of providing AFOs
Version	4
Date	28-11-2013
Coordinating investigator/project leader	[redacted]
Principal investigator(s) (in Dutch: hoofdonderzoeker/uitvoerder)	[redacted]<br>[redacted]
Sponsor (in Dutch: verrichter/opdrachtgever)	Roessingh Research and Development BV.<br>Roessinghsbleekweg 33B, 7522 AH Enschede<br>053-4875777
Independent physician(s)	[redacted]

Laboratory sites	Gait lab Roessingh Research and Development,<br><br>Roessinghsbleekweg 33B, 7522 AH Enschede<br><br>053-4875777

PROTOCOL SIGNATURE SHEET

Name	Signature	Date
Sponsor or legal representative:<br>For non-commercial research,<br>Head of Department:<br>[redacted]		28-11-2013
Coordinating Investigator/Project leader/Principal Investigator:<br>[redacted]		28-11-2013

TABLE OF CONTENTS

1. INTRODUCTION AND RATIONALE 10
2. OBJECTIVES 13
3. STUDY DESIGN 14
4. STUDY POPULATION 16
   4.1 Population (base) 16
   4.2 Inclusion criteria 16
   4.3 Exclusion criteria 16
   4.4 Sample size calculation 17
5. TREATMENT OF SUBJECTS 18
   5.1 Investigational product/treatment 18
6. METHODS 19
   6.1 Study parameters/endpoints 19
      6.1.1 Main study parameter/endpoint 19
      6.1.2 Secondary study parameters/endpoints 20
      6.1.3 Other study parameters 21
   6.2 Randomisation, blinding and treatment allocation 22
   6.3 Study procedures 22
   6.4 Withdrawal of individual subjects 25
      6.4.1 Specific criteria for withdrawal 25
   6.5 Replacement of individual subjects after withdrawal 25
   6.6 Follow-up of subjects withdrawn from treatment 25
   6.7 Premature termination of the study 25
7. SAFETY REPORTING 26
   7.1 Section 10 WMO event 26
   7.2 Adverse and serious adverse events 26
   7.3 Follow-up of adverse events 26
   7.4 Data Safety Monitoring Board (DSMB) 26
8. STATISTICAL ANALYSIS 27
   8.1 Descriptive statistics 27
   8.2 Univariate analysis 27
9. ETHICAL CONSIDERATIONS 28
   9.1 Regulation statement 28
   9.2 Recruitment and consent 28
   9.3 Benefits and risks assessment, group relatedness 28
   9.4 Compensation for injury 29
   9.5 Incentives 29
10. ADMINISTRATIVE ASPECTS AND PUBLICATION 30
   10.1 Handling and storage of data and documents 30
   10.2 Amendments 30
   10.3 Annual progress report 30
   10.4 End of study report 31

10.5 Public disclosure and publication policy 31
11. REFERENCES 32

LIST OF ABBREVIATIONS AND RELEVANT DEFINITIONS

ABR	ABR form (General Assessment and Registration form) is the application form that is required for submission to the accredited Ethics Committee (ABR = Algemene Beoordeling en Registratie)
AE	Adverse Event
AR	Adverse Reaction
CA	Competent Authority
CCMO	Central Committee on Research Involving Human Subjects
CV	Curriculum Vitae
DSMB	Data Safety Monitoring Board
EU	European Union
EudraCT	European drug regulatory affairs Clinical Trials GCP Good Clinical Practice
IB	Investigator’s Brochure
IC	Informed Consent
IMP	Investigational Medicinal Product
IMPD	Investigational Medicinal Product Dossier
METC	Medical research ethics committee (MREC); in Dutch: medisch ethische toetsing commissie (METC)
(S)AE	Serious Adverse Event
SPC	Summary of Product Characteristics (in Dutch: officiële productinfomatie IB1-tekst)
Sponsor	The sponsor is the party that commissions the organisation or performance of the research, for example a pharmaceutical company, academic hospital, scientific organisation or investigator. A party that provides funding for a study but does not commission it is not regarded as the sponsor, but referred to as a subsidising party.
SUSAR	Suspected Unexpected Serious Adverse Reaction
Wbp	Personal Data Protection Act (in Dutch: Wet Bescherming Persoonsgevens)
WMO	Medical Research Involving Human Subjects Act (Wet Medisch-wetenschappelijk Onderzoek met Mensen

SUMMARY

Rationale: Ankle-foot orthoses (AFOs) are often used in the rehabilitation after stroke. Up till now, the effects of AFOs are mainly studied performing cross-over studies to assess the immediate and short-term effects. Evidence for long-term effects, and especially research studying the timing of providing AFOs is lacking. In order to study the effects of timing of providing AFOs after stroke a new longitudinal research is proposed. In this explorative study acute stroke patients will be included and 2 intervention groups will be arranged. Both intervention groups will differ in the moment in which the AFO will be provided during the rehabilitation process.

Objective: The primary objective of this research is to study the effects of timing of providing AFOs on mobility related activities and activities of daily living in acute stroke patients in a rehabilitation setting. Secondary, the effects of timing of providing AFOs on walking impairments, occurrence of falls and fall related events and the effect on quality of life is studied.

Study design: This explorative study has a longitudinal study design with randomisation for the intervention. The first measurements will be performed as soon as possible after admission into the rehabilitation centre. Within the first 6 months after stroke, the measurements will be performed with 2-week/monthly intervals. Follow-up measurements will be performed 12 months after stroke. The effects of timing of providing an AFO will be assessed using gait analysis, instrumented tests, clinical tests and questionnaires.

Study population: Acute stroke patients admitted into the Roessingh Rehabilitation Centre in Enschede will participate. Subjects with an indication to use an AFO because of problems with stability in stance, problems with footclearance during swing or problems with foot prepositioning in early stance will be included.

Intervention: Timing of providing AFOs will be compared in 2 groups: 1) providing an AFO within one week after admission into the rehabilitation centre; and 2) providing an AFO 8 weeks later.

Main study parameters/endpoints: Main study parameters are: mobility related activities and activities of daily living (clinical tests, questionnaires). Secondary, walking impairments (gait analysis, instrumented tests, clinical tests), occurrence of falls and fall related events and quality of life after stroke will be assessed (questionnaires).

Nature and extent of the burden and risks associated with participation, benefit and group relatedness: During the study the subjects will be measured intensively. The first 6 months the measurements will be performed with 2-week/monthly intervals, follow-up measurements will be performed 12 months after stroke. The measurements will be spread throughout the week to minimise the burden for the subjects. During all the measurements,

subjects can take rest any time they like to prevent fatigue. A physiotherapist and/or researcher will accompany the subjects during all measurements.

1. INTRODUCTION AND RATIONALE

Stroke world wide

It was estimated that in 2001 cerebrovascular diseases (stroke) accounted for 5.5 million deaths world wide (World Health Organization, 2002). This is 9.6% of all deaths. Additionally, stroke is the leading cause of disability in adults. Six months after stroke, only 60% of people with initial hemiparesis have achieved functional independence in simple activities of daily living such as toileting and walking short distances (Verheyden et al. 2008). Walking disability after stroke may improve using assistive devices. In case of decreased postural stability canes and walkers are effective devices (Jutai et al. 2007). Furthermore, orthoses are often used to improve walking. An orthosis is defined as an “externally applied device used to modify the structural or functional characteristics of the neuromusculo-skeletal system”. Its primary function is to control abnormal motion of one or more body segment(s) around a joint(s) (Goodwill et al. 1977). In case of ankle-foot orthoses (AFOs), the orthosis can provide lateral stability at the ankle in stance phase, facilitate toe clearance in swing phase and promote heel strike (Leung and Mosely, 2003).

Ankle-foot orthoses

Although AFOs are often used in clinical practice, the literature is inconclusive about the effects of AFOs. Both positive effects (de Wit et al. 2004; Tyson and Thornton, 2001; Simons et al. 2009) and no additional effects of AFOs (Churchill et al. 2003; Gök et al. 2003) on gait parameters and functional outcome measures have been reported. In a recent Cochrane review Tyson et al. analysed 14 trials with 429 participants using orthotic devices after stroke (Tyson and Kent, 2009). The overall effect of lower limb orthoses on walking disability (speed), walking impairment (step/stride length) and balance impairment (weight distribution in standing) was significant and beneficial. The effects on postural sway (balance impairment) and mobility showed a positive trend favouring an AFO, but did not reach statistical significance. All included trials were cross-over trials assessing the immediate, short-term effects of wearing the AFO. Tyson therefore discussed that although immediate effects are relevant, the evidence is of insufficient level to inform clinicians in daily practice because many aspects concerning the long-term effects of using AFOs are still unanswered. One of these aspects concerns the timing of providing AFOs. To the best of our knowledge, there are no studies that evaluated this aspect of AFO use. Answering these questions implicates a switch from cross-over designs to longitudinal intervention studies. Therefore, new research is proposed.

New research

In order to study the effects of timing of providing AFOs in stroke patients a new longitudinal research is proposed. In this study acute stroke patient will be included and 2 intervention groups will be arranged. Both intervention groups differ in the moment in which the AFO will be provided during the rehabilitation process. One intervention group will receive an AFO within one week after admission into the rehabilitation centre. Subjects in the other intervention group will receive an AFO 8 weeks later.

The rational behind the early provision of AFOs is that AFOs are reported to improve a safe walking pattern, for example in case of a foot drop (Geboers et al. 2002). In a recent study stroke patients felt that walking devices (including AFOs) improved their walking, confidence and safety (Tyson and Rogerson, 2009). If patients are able to ambulate at a safer level earlier in the rehabilitation phase due to early prescription of AFOs, patients may become more active while experiencing less falls. This might stimulate rehabilitation, as patients may become more independent in activities of daily life (ADL) in an earlier phase of the rehabilitation, or reach a higher level of ADL-independence at the end of rehabilitation. Other positive effects of early AFO use might be seen on the level of compensation strategies. In case of a dropfoot due to paresis of the m. Tibialis anterior, compensatory circumduction of the hip is often seen in swing phase to overcome the relative long leg length. If AFOs are provided earlier in rehabilitation, those compensatory movements might not be necessary and possibly walking patterns and walking speed will improve.

Besides measuring potential beneficial effects, possible adverse effects and the patients' opinion are important aspects that should be studied when evaluating the effects of using AFOs. In traditional neurodevelopment concepts of stroke physiotherapy AFO use is discouraged as AFOs are suggested to prevent or delay recovery of normal movement (Bobath, 1990). Immobilisation of the ankle may weaken the present muscles around the ankle, thereby inducing disuse and worsening the effect of loss of muscle strength. However, in a study by Geboers et al. immediate and long-term effects of AFOs on muscle activity during walking were studied in subjects with foot drop (Geboers et al. 2002). They concluded that AFO use by these recently paretic patients (peripheral paresis, 6 weeks to 1 year) did not seem to induce significant changes in muscle reaction after 6 weeks of daily use, compared with the immediate reaction. Therefore, they stated that no contraindications exist for AFO use in recent paretic subjects to enhance safe walking. Whether these effects also account for paresis after stroke is not clear. In addition to the discussion about the possible delaying effects of AFOs on recovery of normal movement, Tyson et al. studied the patients opinion of using walking aids in non-ambulant acute stroke patients (Tyson and Rogerson, 2009). In a randomised controlled trial no assistive device, a walking cane, an AFO, a slider shoe, and walking with each of the devices was compared. When asked, 70% of the subjects would rather walk now using an assistive device than delay walking and learn to walk with a normative gait pattern without a device.

ICF

The proposed research includes a wide scale of measurements to be performed, in order to study a range of aspects concerning the effects of timing of providing AFOs after stroke. When selecting the specific parameters and clinical tests, the framework of the World Health Organization, the International Classification of Functioning (ICF) model was used (Nederlands WHO-FIC Collaborating Centre, 2002). The ICF distinguishes the levels "body functions and structures" to define impairments, the level of "activity" to define disabilities and the "participation" level to define handicaps. In the proposed study outcome measures will mainly focus on the ICF levels of body function and activity.

In summary, a new explorative longitudinal research is proposed, in which the effects of timing of using an AFO during rehabilitation of acute stroke patients will be studied. Providing AFOs within one week after admission will be compared with providing AFOs later on in the rehabilitation process. Outcome measures mainly focus on the ICF levels “body functions” and “activity”.

2. OBJECTIVES

Primary objective:

- What are the effects of timing of providing ankle-foot orthoses on mobility related activities and activities of daily living in acute stroke patients in a rehabilitation setting?

  Hypothesis:
  Compared to late provision, early provision of AFOs is expected to positively affect mobility and activities of daily life after stroke. Walking independence, walking speed and endurance are expected to improve. Furthermore, independence in activities of daily life is expected to improve.

Secondary objective:

- What are the effects of timing of providing ankle-foot orthoses on walking impairments in acute stroke patients in a rehabilitation setting?

  Hypothesis:
  Early provision of AFOs (± 2 wks after stroke) positively affects walking impairments after stroke compared to providing AFOs later on in the rehabilitation process (± 10 wks after stroke), as the development of compensatory movements in the proximal segments of the lower limb and compensatory muscle patterns during walking are expected to diminish.

- What are the effects of timing of providing ankle-foot orthoses on the occurrence of falls and fall related events in acute stroke patients in a rehabilitation setting?

  Hypothesis:
  Compared to late provision, early provision of AFOs is expected to diminish the number of falls and fall related events as AFOs are expected to improve safe ambulation and diminish fear to fall.

- What are the effects of timing of providing ankle-foot orthoses on quality of life in acute stroke patients in a rehabilitation setting?

  Hypothesis:
  Compared to late provision, early provision of ankle-foot orthoses is expected to improve quality of life after stroke as AFOs are expected to improve the level of independence in performing daily life activities.

3. STUDY DESIGN

In this explorative study a randomised experimental longitudinal design will be used. The intervention concerns the timing of providing AFOs to acute stroke patients during the rehabilitation process. In two groups the effect of timing of providing AFOs will be compared: 1) providing an AFO within one week after admission in the rehabilitation centre (± 2 weeks after stroke); and 2) providing an AFO 8 weeks later.

Acute stroke subjects with indication for using an AFO during rehabilitation will be included. To avoid differences in therapy treatment and intensity between subjects, only one rehabilitation centre will participate in this study; The Roessingh Rehabilitation Centre in Enschede. Patients will be included in the study as soon as possible after admission into the Roessingh. In daily practice, this is within approximately 14 days after stroke onset. Participating patients will be randomised into 2 groups. The first group will receive the AFO within one week after admission into the Roessingh (referred to as the “early” AFO group). Subjects in the “early” group can practise walking and transferring using the AFO all day long: during the physiotherapy sessions, during other therapy sessions and during the stay at the ward. The second group (referred to as the “late” AFO group) will receive the AFO 8 weeks later than the “early” AFO group, approximately 10 weeks after stroke. In the period before receiving the AFO, this group will practise walking and transferring without support of the ankle and foot. If, during physiotherapy sessions, practising without support of the ankle appears to be impossible, bandaging of the ankle and foot is allowed, but only during the physiotherapy sessions. This bandaging is not continued during other therapy sessions, or during the stay at the ward.

The “late” AFO group will receive the AFO approximately 10 weeks after stroke. This period is chosen for 2 reasons. First, this is comparable to the period in which in clinical practice the final AFO is usually provided according to physiotherapists at the Roessingh (approximately 8-12 weeks after stroke). Second, prospective stroke studies indicate that the process of spontaneous recovery after stroke is almost completed after approximately 6-10 weeks (Buurke et al. 2008; Kwakkel et al. 2006).

In a longitudinal study the included subjects will be assessed repeatedly during the first year after stroke. In order to measure the effects of timing of providing an AFO, the ICF levels activity and function will be used. On the ICF level “function”, gait analysis, instrumented tests and various clinical tests will be performed. On the level of “activities”, several clinical tests will be assessed (see paragraph 6.1). Furthermore, the incidence of falls and quality of

life after stroke will be assessed using questionnaires. The first measurements will be performed as soon as possible after admission into the Roessingh. Within the first 6 months after stroke, the various measurements will be performed with 2-week/monthly intervals (see paragraph 6.3). Follow-up measurements will be performed 1 year after stroke. The measurements will be performed at the Roessingh Rehabilitation Centre, and in the gait laboratory at the Roessingh Research and Development, Enschede.

Flowchart:

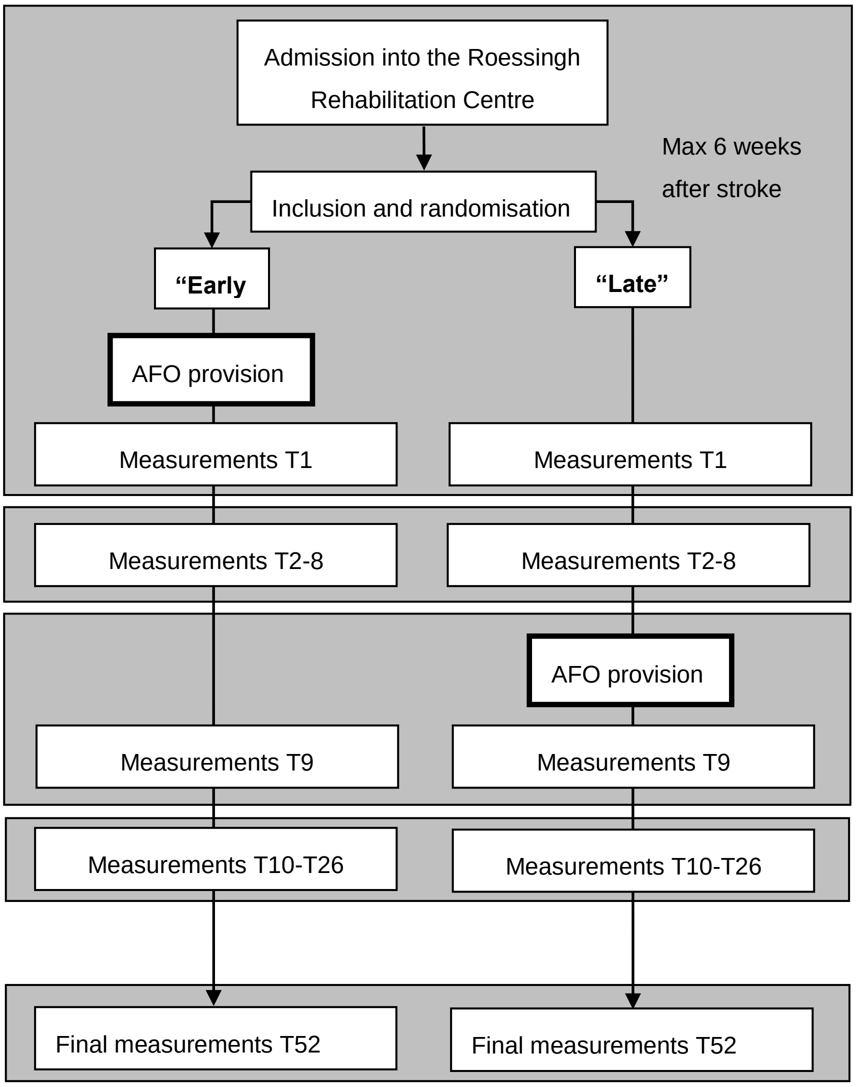

T1 = week of inclusion and providing AFO to "early" group;
T9 = week of providing AFO to "late" group

4. STUDY POPULATION

4.1 Population (base)

Acute stroke patients will be recruited from the Roessingh Rehabilitation Centre. Rehabilitation physicians from the Roessingh Rehabilitation Centre ([redacted]), all experienced in the management of stroke patients and previous involved in research projects, will carry out the first step in the subject recruitment. The rehabilitation physicians will select the subjects after admission into the Roessingh Rehabilitation Centre and ask them if they are interested in taking part in a research studying the effects of timing of providing an ankle-foot orthosis after stroke. If so, the physician will ask them if they give permission to pass on their names to the responsible researcher at the Roessingh Research and Development (RRD). The researcher will contact the patient and will provide hem/her and his/her relatives, both verbally as well as written, with the necessary information about the study. In the information letter details about the purpose of the study and the measurements that will be performed are explained. All subjects are informed that they can stop with the measurements at any moment, without giving any reason and without experiencing any disadvantage.

4.2 Inclusion criteria

- admission into the Roessingh Rehabilitation Centre
- unilateral ischaemic or haemorrhagic hemiparetic stroke (single and first ever or history of previous stroke with full physical recovery)
- time since stroke max 6 weeks
- age 18 years or older
- indication to use an AFO during rehabilitation because of:
    - problems with stability in stance
    - problems with footclearance during swing
    - problems with foot prepositioning in early stance
- no severe deficits in communication
- no severe deficits in memory and understanding

4.3 Exclusion criteria

- medical history of previous stroke(s) without full physical recovery
- complicating medical history such as cardiac, pulmonary, or orthopaedic disorders that could affect performance of the included measurements
- severely impaired sensation
- suffering from severe neglect
- suffering from comprehensive aphasia
- participation in other scientific research

4.4 Sample size calculation

To our knowledge this is the first explorative research studying the effects of timing of AFOs in the rehabilitation after stroke. As such, data of earlier experiments with timing of AFOs are not available for power calculation.

The number of subjects that can be included in the study will depend on the number of acute stroke patients admitted to the rehabilitation centre. Based on estimations of therapists and rehabilitation physicians of the Roessingh Rehabilitation Centre and the Annual Reports of the Roessingh, we estimate that approximately 20 subjects can be included each year. The time during which subjects can be included is defined at 30 months (starting from September 2009). Within the available inclusion time of 30 months therefore 50 subjects can be included.

An interim power-analysis will be performed after including 30 subject to estimate whether including 50 subjects will be enough to reach sufficient statistical power. If this interim analysis reveals that more than the planned 50 subjects are needed, an amendment will be applied to include more subjects.

5. TREATMENT OF SUBJECTS

5.1 Investigational product/treatment

Acute stroke patients with indication for using an AFO will be included in this study. The intervention of interest is the timing of providing an AFO during the rehabilitation process. Early provision of AFOs (within one week after admission into the rehabilitation centre), will be compared with providing an AFO 8 weeks later.

There are several types of AFOs that are used in daily practice. Three commonly used types of AFOs are selected for this study (see pictures below). With the selected types of AFOs both patients with problems during the swing and stance phase of walking can be provided with an AFO. The so called “Dynafo” and “Ottobock” orthoses are flexible types of orthoses. They allow some dorsiflexion and vertical rotation movement in the ankle and are primarily used to correct a dropfoot and small varus deformities of the ankle and foot during the swing phase of walking. In case a higher degree of stability is needed, for example to counteract hyperextension of the knee in stance, or to correct varus deformity at the ankle in stance and/or swing due to spasticity, the “Camp” orthosis will be used. This is a more rigid type of AFO, which surrounds most of the calf and both malleoli, and therefore restricts most movement in the ankle. All AFOs are fixed with a band around the lower leg and are worn inside the shoe with a sole plate that extents below the foot. The selected AFOs can be provided for the left or right lower limb and are fabricated in various sizes. If necessary, for example in case of sensation disorders, the sole plate will be customized.

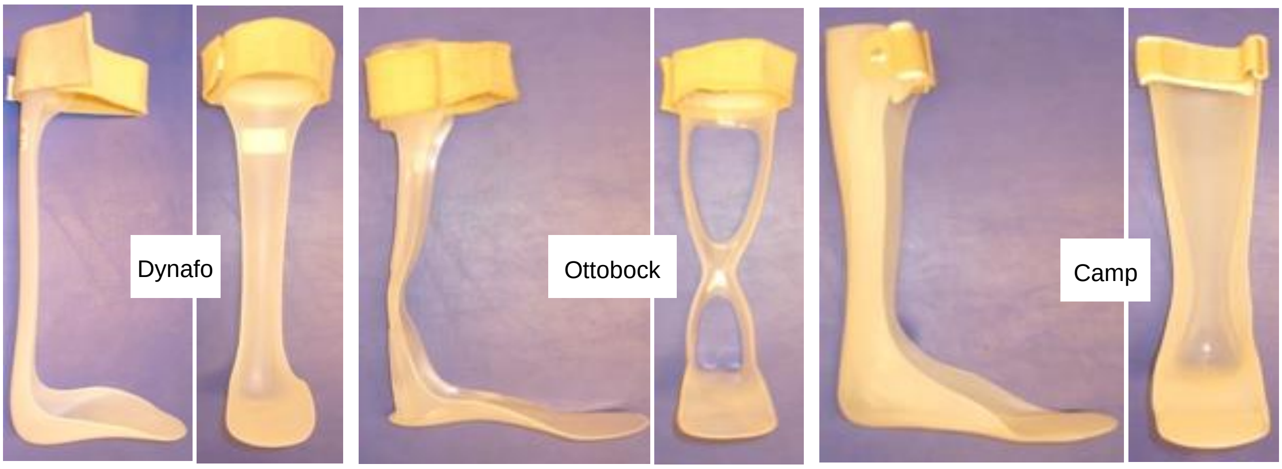

6. METHODS

6.1 Study parameters/endpoints

6.1.1 Main study parameter/endpoint

In this study the effects of early provision of ankle-foot orthoses will primarily be assessed on the level of the ICF level “activities”. The included parameters (see below) will assess the effects on the patient’s mobility and daily life activities.

Mobility related activities and activities of daily living

On the ICF level “activities” the effect of timing of providing AFOs is assessed on mobility and activities of daily life. Therefore, several clinical tests are used: 10m walking test (Wade, 1992), 6min walking test, Functional Ambulation Categories (Holden et al. 1984), Timed Up&Go test (Podsiadlo and Richardson, 1991), Stairstest (de Wit et al. 2004), Rivermead Mobility Index (Collen et al. 1991), Berg Balance Scale (Berg et al. 1989), Barthel Index (Collin et al. 1988), Nottingham extended ADL - index (Harwood and Ebrahim, 2002).

Walking velocity:

- 10m walking test (10MWT): comfortable walking speed is measured over a distance of 10m. If necessary walking aids are allowed, physical help is not allowed. Walking speed is calculated by averaging the results of 3 performances.

Walking endurance:

- 6min walking test (6MWT): Comfortable walking speed is measured during 6 minutes. The distance covered in 6 minutes is measured. In case the subject stops before the 6 minutes are over, time and distance are reported.

Independence of walking:

- Functional Ambulation Categories (FAC): evaluates the degree of independence in walking. Performance is graded on a 6-point scale, with higher scores reflecting increasing independence of walking.

Mobility:

- Timed Up&Go test (TUG): The examiner times the subject as he/she performs the following activity: from a sitting position in a standard-height armchair, the subject independently stands up, walks 3 meters (with assistive device as needed), turns around, walks back and sits down again. The average of 3 tests is calculated.
- Stairstest (ST): assesses mobility like the TUG, with addition of 2 tasks: stairs ascent and descent. Patients are timed while rise from a chair, walk and ascend a flight of 12 stairs, walk and touch a

wall, turn around, descend the stairs, walk back to the chair and sit down again. The average time of 3 tests is calculated.

- Rivermead Mobility Index (RMI): a questionnaire with 15 items concerning mobility, graded on a 2-point scale.

Balance:

- Berg Balance Scale (BBS): evaluates balance and is composed of 14 balance tasks that require the subject to maintain a static position, change the orientation of the centre of mass with respect to the base of support and diminish the base of support. Performance of each task is graded from 0 (cannot perform) to 4 (normal performance).

Activities of daily life:

- Barthel Index (BI): evaluates the level of independence during activities of daily life. Ten items are evaluated on a 2/3/4-point scale.
- Nottingham Extended ADL – Index (NEADL): evaluates the level of independence during extended activities of daily life. Twenty-two items are evaluated on a 4-point scale.

6.1.2 Secondary study parameters/endpoints

When the effects of early provision of AFOs only measured only on the activity level, one cannot differentiate whether changes appear because of restoration of the paretic side or because of compensation by the non-paretic side. Therefore, secondly the effects of early AFO provision will be assessed of the ICF level "body function". During gait analysis (see below) both the paretic and non-paretic side will be measured in order to explain changes on the activity level.

<u>Walking impairment</u>

Walking impairment (ICF level: body function) will be assessed during gait analysis measuring kinetics (ground reaction forces), 3D kinematics (joint angles) and temporal and spatial parameters of the walking pattern (step length, step time, step width, single/double stance time). Simultaneously, muscle activity from m. Rectus femoris, m. Vastus lateralis, m. Semitendinosus, m. Gastrocnemius and m. Tibialis anterior of both legs will be recorded using surface electromyography (EMG). Subjects will be measured while walking at comfortable speed, with and without AFO. If necessary, subjects are allowed to use walking aids like a cane.

Besides using gait analysis, body functions will be assed using an instrumented test (Mappam procedure (Fleuren et al. 2006)) and clinical tests: the Motricity Index (Collin and Wade, 1990), Modified Ashworth Scale (Bohannon and Smith, 1987), Brunnstrom Fugl Meyer (leg portion, Fugl-Meyer et al. 1975), passive ankle angles (Ryf and Weymann, 1999).

- Mappam procedure: instrumented test to measure stretch reflex activity of the knee flexor and extensor muscles with surface EMG during the pendulum test (passive swing movement of the lower leg due to gravity) and during passive and active movement of the lower leg. Furthermore, the Visual Analog Scale (VAS-scale) will be used to assess the perception of spasticity and spasticity-related discomfort.
- Motricity Index (MI, leg portion): evaluates selective muscle control and maximal isometric muscle contraction of the lower limb, graded on a 6-point scale.
- Modified Ashworth Scale (MAS): evaluates the resistance to passive plantar and dorsal flexion in the ankle, graded on a graded on a 6-point scale.
- Brunnstrom Fugl Meyer assessment (BFM, leg portion): evaluates the amount of synergies in the lower limb, graded on a 3-point scale.
- Ankle angles (dorsiflexion, plantarflexion) during passive movement.

As secondary parameters fear of falling (Falls Efficacy Scale (Hellstrom and Lindmark, 1999), occurrence of falls and quality of life after stroke (Stroke Impact Scale, van der Port et al. 2008) are measured.

Falling:

- Falls Efficacy Scale (FES): evaluates the experienced confidence of the subject, and thereby the fear to fall, during activities of daily life, like walking outdoors or walking the stairs. Thirteen items are scored on a 11-point scale.

The occurrence of falls and all fall related events are measured using questionnaires for therapists and subjects (see attachment 1).

Quality of life:

- Stroke Impact Scale (SIS version 3.0): evaluates the effects of stroke in terms of function, activities and participation. Fifty-nine items are scored on a 5-point scale

6.1.3 Other study parameters

At the start of the study the following subject characteristics will be measured:

- sex
- age (years)

- weight (kg)
- height (m)
- time since stroke (weeks)
- affected side (left/right)
- stroke type (ischaemic or haemorrhagic)
- dominant side (left/right)
- propriosepsis and sensation disorders (Modified Nottingham Sensory Assessment, Lincoln et al. 1998)
- cognition (Mini-Mental State Examination, Folstein et al. 1975)
- communication level (spontane communicatie onderdeel, Utrecht Communicatie Onderzoek, Blauw and Koning, 1988)

The characteristics of the treatment of the individual subject will be measured using questionnaires for clinicians and subjects (see attachment 1):

- use of additional walking aids
- standing and walking
- falling
- medication (spasticity related)
- AFO use

Furthermore, a questionnaire will be used to assess pain of the feet/ankles and using the shoes and AFO (see attachment 2).

6.2 Randomisation, blinding and treatment allocation

The assignment to the “early or “late” AFO group will be randomised using block randomisation with blocks of 4 subjects. An independent person will perform this randomisation using random numbers (Fisher). Unfortunately, blinding of the patients and/or assessors is not possible because it is not possible to mask whether or not someone is wearing an AFO.

6.3 Study procedures

The Modified Nottingham Sensory Assessment (propriosepsis and sensation), Mini-Mental State Examination (cognition) and the Utrecht Communicatie Onderzoek (communication level) are used to describe the subject population. These standard tests are performed once, in the first weeks after inclusion.

Gait analysis

Gait analysis will be performed at the RRD. Both groups will be measured five times (see table 1):

T1: The first measurement will be performed at the end of the first week of the study (T1). Subjects in the "early" AFO group receive their AFO at the beginning of this week and are measured with and without AFO. Subjects in the "late" AFO group will be measured without AFO.

T9: The second gait analysis will be performed in week 9 of the study (T9). Subjects in the "early" group are now using the AFO for 8 weeks, subjects in the "late" group receive their AFO in this week. From now on both groups will be measured with and without using the AFO.

T17 The third gait analysis will be performed in week 17 of the study (T17). Subjects in the "early" group are using their AFO for 16 weeks, subjects in the "late" group are using their AFO 8 weeks.

T26 The fourth gait analysis will be performed in week 26 of the study, approximately 6 months after stroke.

T52 The last measurement will be performed 1 year after stroke.

Measurements will be performed while walking at comfortable speed. If necessary, subjects can use walking aids (e.g. canes). The movements of the body while walking will be recorded with a VICON video based 3D kinematic measurement system (VICON-MX13+, Vicon Oxford Metrics, Oxford, UK), consisting of 6 infrared cameras, using passive reflexive markers. Subjects are asked to put on a short and top so the skin is suitable for proper marker placement. These markers will be attached to the subject on the feet, lateral malleolus, tibia, knee and femur of both legs, as well as on the pelvis. Vertical ground reaction forces will be measured with force plates, covered in the level walkway (AMTI-OR6-5, Advanced Medical Technologies, Inc, Massachusetts, USA). During the gait analysis muscle activity will be recorded using surface EMG from several superficial muscles of both legs using wet-gel silver/silver-chloride electrodes. Signals will be collected from the m. Rectus Femoris, m. Vastus Lateralis, m. Semitendinosus, m. Gastrocnemius and m. Tibialis Anterior. Electrode placement, skin preparation and related issues are performed according to the guidelines presented by SENIAM (Hermens et al. 1999).

Clinical tests

All clinical tests and questionnaires measuring walking impairments, mobility, activities of daily life, falling and quality of life are performed according to the specific test protocols. During the first 18 weeks of the study most clinical tests will be repeated with 2-week intervals. Furthermore, the tests will be repeated 6 and 12 months after stroke (T26 and T52, respectively). All the clinical tests are performed with AFO.

The frequency of performing the various measurements is listed in Table 1.

Spasticity of the lower limb is assessed using the Mappam procedure. The subject is in a comfortable semi-reclined position (45º) with support for the back and lumbar region. Knee flexor and extensor muscles are recorded while performing the pendulum test (passive swing movement of the lower leg from full extension and than released), and during passive movement. The subject is instructed to relax his/her leg and not to oppose or facilitate the passive movements. The subject is asked to perform an active movement. The knee joint angle is measured with an electric goniometer placed on the lateral side of the knee. Surface EMG records the activity of the m. Rectus femoris and the Semitendinosus muscles. Electrode placement, skin preparation and related issues are performed according to the guidelines presented by SENIAM (Hermens et al. 1999).

Table 1: Measurements to be performed

Period	Measurement	Content	
T1	Modified Nottingham Sensory Ass<br>Mini-Mental State Examination<br>Utrecht Communicatie Onderzoek	propriosepsis and sensation<br>cognition<br>communication	*<br>**<br>***
T1, T9, T17, T26, T52	Clinical Gait analysis<br>Mappam test<br>Modified Ashworth Scale<br>Brunnstrom Fugl Meyer (leg portion)<br>Passive ankle range<br>Barthel Index<br>Nottingham Extended ADL (T26/52)<br>Stroke Impact Scale	joint angles/EMG/GRF<br>stretch reflex, instrumented<br>resistance to passive<br>motion<br>synergies<br>passive range of motion<br>ADL<br>extended ADL<br>quality of life after stroke	*<br>*<br>*<br>*<br>*<br>**<br>**<br>**
T1-T18: 2-week interval, T26, T52	Motricity Index (leg portion)<br>10-m walking test<br>6-min walking test<br>Berg Balance Scale<br>Timed Up and Go test<br>Stairstest<br>Rivermead Mobility Index<br>Functional Ambulation Categories<br>therapist- and subject questionnaires (attachment 1)	muscle control and strength<br>walking speed<br>walking endurance<br>balance<br>mobility<br>mobility<br>mobility<br>independence in walking<br>treatment and falls	*<br>*<br>*<br>*<br>*<br>*<br>**<br>***<br>**
T1-T26: every month, T52	Falls Efficacy Scale<br>subject questionnaire (attachment 2)	fear of falling<br>pain and AFO/shoe use	**<br>**

T1 = week of inclusion and providing AFO to the “early” group;

T9 = week of providing AFO to the “late” group;

T26 = 6 months after stroke

T52 = 12 months after stroke

) physical test; **) questionnaire; *)rating scale*

6.4 Withdrawal of individual subjects

Subjects can leave the study at any time for any reason if they wish to do so without any consequences. The investigator can decide to withdraw a subject from the study for urgent medical reasons.

6.4.1 Specific criteria for withdrawal

Subjects will be withdrawn when either the subject him/herself or the researcher indicates that the load of the measurements is too much.

6.5 Replacement of individual subjects after withdrawal

Individuals withdrawn from the study will be replaced by new subjects using “modified intention to treat analysis” in case individuals are withdrawn before start of the intervention (using an AFO).

6.6 Follow-up of subjects withdrawn from treatment

No follow-up will take place after subjects have been withdrawn from the study.

6.7 Premature termination of the study

In case of serious adverse events (see paragraph 7.2) the study can be terminated prematurely. In such circumstances, the accredited METC will be notified and the subjects will be informed as soon as possible.

7. SAFETY REPORTING

7.1 Section 10 WMO event

In accordance to section 10, subsection 1, of the WMO, the investigator will inform the subjects and the reviewing accredited METC if anything occurs, on the basis of which it appears that the disadvantages of participation may be significantly greater than was foreseen in the research proposal. The study will be suspended pending further review by the accredited METC, except insofar as suspension would jeopardise the subjects' health. The investigator will take care that all subjects are kept informed.

7.2 Adverse and serious adverse events

Adverse events are defined as any undesirable experience occurring to a subject during a clinical trial. All adverse events reported spontaneously by the subject or observed by the investigator or his staff will be recorded.

A serious adverse event is any untoward medical occurrence or effect that at any dose results in death;

- is life threatening (at the time of the event);
- requires hospitalisation or prolongation of existing inpatients' hospitalisation;
- results in persistent or significant disability or incapacity;
- is a congenital anomaly or birth defect;
- is a new event of the trial likely to affect the safety of the subjects, such as an unexpected outcome of an adverse reaction, lack of efficacy of an IMP used for the treatment of a life threatening disease, major safety finding from a newly completed animal study, etc.

All SAEs will be reported to the accredited METC that approved the protocol, according to the requirements of that METC.

7.3 Follow-up of adverse events

All adverse events will be followed until they have abated, or until a stable situation has been reached. Depending on the event, follow up may require additional tests or medical procedures as indicated, and/or referral to the general physician or a medical specialist.

7.4 Data Safety Monitoring Board (DSMB)

No DSMB has been established.

8. STATISTICAL ANALYSIS

8.1 Descriptive statistics

Descriptive statistical methods are applied for each of the outcome measures. Presentation of data is done by calculation of mean ± standard deviation (SD), range and frequency. Furthermore, descriptive statistics are used to describe the subject and treatment characteristics (paragraph 6.1.3).

8.2 Univariate analysis

A significance level of $\alpha = 0.05$ will be used in data analysis. If necessary, adjustments for multiple comparisons will be applied.

Analysis of this study consists of studying the effects of timing of providing AFOs on the outcome measures. Comparison of the data between the “early” and “late” AFO group will be performed using corresponding parametric/non-parametric tests according to the features of the outcome measures involved (t-test).

9. ETHICAL CONSIDERATIONS

9.1 Regulation statement

This study will be conducted according to the principles of the Declaration of Helsinki (52nd WMA General Assembly, Edinburgh, Scotland, October 2000, Note of Clarification added by the WMA General Assembly, Tokyo 2004) and in accordance with the Medical Research Involving Human Subjects Act (WMO).

9.2 Recruitment and consent

The rehabilitation physicians from the Roessingh Rehabilitation Centre ([redacted]) will approach the stroke patients when admitted to the rehabilitation centre. They will first ask patients who meet the in- and exclusion criteria if he/she is interested to participate in a study about timing of AFOs. If so, they will ask if they give permission to pass on their names to the responsible researcher at the RRD. The researcher will provide verbal and written information about the study. In the information letter details about the purpose of the study and the measurements will be given. Patients have 2 days to consider their participation. After a positive answer the subject will be asked to sign the informed consent and the first measurements will be performed.

9.3 Benefits and risks assessment, group relatedness

A physiotherapist and a researcher (both experienced in measuring stroke patients) will be responsible for carrying out the measurements.

Subjects may lose their balance during gait analysis or while performing the clinical tests. The chance that performing these tasks will lead to a fall is not higher than during usual rehabilitation, since the tasks that are performed resemble tasks being practised during rehabilitation. If necessary, subject are allowed to use walking aids like a cane during most of the tests. During all measurements a physiotherapist or researcher will accompany the subjects.

The research protocol includes a list of measurements which have to be performed on a regular basis. Some of the included tests (like the BBS, 10-m walking test and 6-min walking test) are also performed in usual rehabilitation care, although not in a 2-weekly

interval. The measurements will be spread throughout the week to minimise the burden for the subjects. During all the measurements, subjects can take rest any time they like to prevent fatigue.

In the past, no previous studies are performed comparing "early" provision of AFOs with providing AFOs later on in the rehabilitation process. Therefore, no clear risks or benefits for the intervention groups can be specified. Potential benefits (like improving independence in daily life activities, improving compensatory movements during walking) and risks (like interfering in the recovery of normal movement) are previously discussed in the introduction.

9.4 Compensation for injury

The sponsor/investigator has a liability insurance which is in accordance with article 7, subsection 6 of the WMO.

The sponsor (also) has an insurance which is in accordance with the legal requirements in the Netherlands (Article 7 WMO and the Measure regarding Compulsory Insurance for Clinical Research in Humans of 23th June 2003). This insurance provides cover for damage to research subjects through injury or death caused by the study.

1. € 450.000,-- (i.e. four hundred and fifty thousand Euro) for death or injury for each subject who participates in the Research;
2. € 3.500.000,-- (i.e. three million five hundred thousand Euro) for death or injury for all subjects who participate in the Research;
3. € 5.000.000,-- (i.e. five million Euro) for the total damage incurred by the organisation for all damage disclosed by scientific research for the Sponsor as 'verrichter' in the meaning of said Act in each year of insurance coverage.

The insurance applies to the damage that becomes apparent during the study or within 4 years after the end of the study.

9.5 Incentives

If the participant makes any travelling costs due to participation of the research, travelling-allowance will be made.

10. ADMINISTRATIVE ASPECTS AND PUBLICATION

10.1 Handling and storage of data and documents

The data analysis and –reportage will be performed in consideration of the guidelines for privacy protection. The personal data will be handled confidentially. The data are filled in on separate forms, where only a personal, not reducible number assigned to the particular subject appears; not a name. The data from the used questionnaires will also be related to the unique number that is attached to the participant. In case it is necessary to be able to trace data to an individual participant, a participant identification code lists is used to link the data to the individual participant. The key to the code is safeguarded by the principal investigator. The data will only be used for statistical and scientific purposes and the participant gives their permission to do so, according to the informed consent. In publications no subject names will be used.

10.2 Amendments

Amendments are changes made to the research after a favourable opinion by the accredited METC has been given. All amendments will be notified to the METC that gave a favourable opinion.

Non-substantial amendments will not be notified to the accredited METC and the competent authority, but will be recorded and filed by the sponsor.
Examples of non-substantial amendments are typing errors and administrative changes like changes in names, telephone numbers and other contact details of involved persons mentioned in the submitted study documentation.

10.3 Annual progress report

The sponsor/investigator will submit a summary of the progress of the trial to the accredited METC once a year. Information will be provided on the date of inclusion of the first subject, numbers of subjects included and numbers of subjects that have completed the trial, serious adverse events/ serious adverse reactions, other problems, and amendments.

10.4 End of study report

The investigator will notify the accredited METC of the end of the study within a period of 8 weeks. The end of the study is defined as the last patient's last visit.

In case the study is ended prematurely, the investigator will notify the accredited METC, including the reasons for the premature termination.

Within one year after the end of the study, the investigator/sponsor will submit a final study report with the results of the study, including any publications/abstracts of the study, to the accredited METC.

10.5 Public disclosure and publication policy

The results of the study will be disclosed in a public trial register

11. REFERENCES

World Health Organization (2002) The World Health Report: 2002. Reducing risks, promoting healthy life.

Verheyden, G., Nieuwboer, A., De Wit, L., Thijs, V., Dobbelaere, J., Devos, H., Severijns, D., Vanbeveren, S. and De Weerdt, W. (2008) Time course of trunk, arm, leg, and functional recovery after ischemic stroke. *Neurorehabil Neural Repair* 22, 173-9.

Jutai, J., Coulson, S., Teasell, R., Bayley, M., Garland, J., Mayo, N. and Wood-Dauphinee, S. (2007) Mobility assistive device utilization in a prospective study of patients with first-ever stroke. *Arch Phys Med Rehabil* 88, 1268-75.

Goodwill, C.J., Chamerlain, M.A. and Evans, C. (1977) Rehabilitation of the physically disabled adult. In: Ponton, F.T., (Ed.) *Orthotics*, pp. 657-684. Cheltenham: Stanley Thornes Ltd]

Leung, J. and Mosely, A. (2003) Impact of Ankle-foot Orthoses on Gait and Leg Muscle Activity in Adults with Hemiplegia. *Physiotherapy* 89, 39-55.

de Wit, D.C., Buurke, J.H., Nijlant, J.M., IJzerman, M.J. and Hermens, H.J. (2004) The effect of an ankle-foot orthosis on walking ability in chronic stroke patients: a randomized controlled trial. *Clin Rehabil* 18, 550-7.

Tyson, S.F. and Thornton, H.A. (2001) The effect of a hinged ankle foot orthosis on hemiplegic gait: objective measures and users' opinions. *Clin Rehabil* 15, 53-8.

Simons, C.D.M., van Asseldonk, E.H.F., van der Kooij, H., Geurts, A.C.H. and Buurke, J.H. Ankle-foot orthoses after stroke: effects on functional balance, weight-bearing asymmetry and the contribution of each lower limb to balance control. *Clinical Biomechanics,* in press

Churchill, A.J., Halligan, P.W. and Wade, D.T. (2003) Relative contribution of footwear to the efficacy of ankle-foot orthoses. *Clin Rehabil* 17, 553-7.

Gök, H., Küçükdeveci, A., Altinkaynak, H., Yavuzer, G. and Ergin, S. (2003) Effects of ankle-foot orthoses on hemiparetic gait. *Clin Rehabil* 17, 137-9.

Tyson, S.F. and Kent, R.M. (2009) Orthotic devices after stroke and other non-progressive brain lesions. *Cochrane Database Syst Rev* CD003694

Geboers, J.F., Drost, M.R., Spaans, F., Kuipers, H. and Seelen, H.A. (2002) Immediate and long-term effects of ankle-foot orthosis on muscle activity during walking: a randomized study of patients with unilateral foot drop. *rch Phys Med Rehabil* 83, 240-5.

Tyson, S.F. and Rogerson, L. (2009) Assistive walking devices in nonambulant patients undergoing rehabilitation after stroke: the effects on functional mobility, walking impairments, and patients' opinion. *Arch Phys Med Rehabil* 90, 475-9.

Bobath, B. (1990) *Adult hemiplegia: evaluation and treatment*, 3rd edition edn. Oxford: Heinemann Medical Books.

Nederlands WHO-FIC Collaborating Centre (2002) *Nederlandse vertaling 'International Classification of Functioning, Disability and Health'*, 1e druk edn. Bohn Stafleu Van Loghum.

Buurke, J.H., Nene, A.V., Kwakkel, G., Erren-Wolters, V., Ijzerman, M.J. and Hermens, H.J. (2008) Recovery of gait after stroke: what changes? *Neurorehabil Neural Repair* 22, 676-83.

Kwakkel, G., Kollen, B. and Twisk, J. (2006) Impact of time on improvement of outcome after stroke. *Stroke* 37, 2348-53.

Fleuren, J., Voerman, G., Nene, A., Snoek, G. and Hermens, H. (2006) Protocol for spasticity assessment in patients with an extracorporeal pumb for intrathecal baclofen (ITB). Roessingh Research and Development.

Collin, C. and Wade, D. (1990) Assessing motor impairment after stroke: a pilot reliability study. *J Neurol Neurosurg Psychiatry* 53, 576-9.

Bohannon, R.W. and Smith, M.B. (1987) Interrater reliability of a modified Ashworth scale of muscle spasticity. *Phys Ther* 67, 206-7.

Fugl-Meyer, A.R., Jaasko, L., Leyman, I., Olsson, S. and Steglind, S. (1975) The post-stroke hemiplegic patient. A method for evaluation of physical performance. *Scand J Rehabil Med* 7, 13-31.

Ryf, C. and Weymann, A. (1999) *Range of Motion - Neutral-0-Method; measurement and documentation.*, Stutgart: Thieme.

Wade, D.T. (1992) *Measurements in neurological rehabilitation*, edn. Oxford: Oxford University Press.

Holden, M.K., Gill, K.M., Magliozzi, M.R., Nathan, J. and Piehl-Baker, L. (1984) Clinical gait assessment in the neurologically impaired. Reliability and meaningfulness. *Phys Ther* 64, 35-40.

Podsiadlo, D. and Richardson, S. (1991) The timed "Up & Go": a test of basic functional mobility for frail elderly persons. *J Am Geriatr Soc* 39, 142-8.

Collen, F.M., Wade, D.T., Robb, G.F. and Bradshaw, C.M. (1991) The Rivermead Mobility Index: a further development of the Rivermead Motor Assessment. *Int Disabil Stud* 13, 50-4.

Berg, K., Wood-Dauphinee, S., Williams, J.I. and Gayton, D. (1989) Measuring balance in the elderly: preliminary development of an instrument. *Physiotherapy Canada* 41, 304-11.

Collin, C., Wade, D.T., Davies, S. and Horne, V. (1988) The Barthel ADL Index: a reliability

study. *Int Disabil Stud* 10, 61-3.

Harwood, R.H. and Ebrahim, S. (2002) The validity, reliability and responsiveness of the Nottingham Extended Activities of Daily Living scale in patients undergoing total hip replacement. *Disabil Rehabil* 24, 371-7.

Hellstrom, K. and Lindmark, B. (1999) Fear of falling in patients with stroke: a reliability study. *Clin Rehabil* 13, 509-17.

van der Port, I.G.L., Leenes, K., Sellmeijer, D. , Zuidgeest, A. and Kwakkel, G. (2008) Betrouwbaarheid en concurrente validiteit van de Nederlandse Stroke Impact Scale 2.0 bij patiënten met een CVA. *Ned Tijdschr Fysiother* 118, 12-18.

Lincoln, N.B., Jackson, J.M. and Adams, S.A. (1998) Reliability and revision of the Nottingham sensory assessment for stroke patients. *Physiotherapy* 84, 358-65.

Folstein, M.F., Folstein, S.E. and McHugh, P.R. (1975) "Mini-mental state". A practical method for grading the cognitive state of patients for the clinician. *J Psychiatr Res* 12, 189-98.

Blauw, M. and Koning, M. (1988) *Afasie, een mulidisciplinaire benadering*, Loosdrecht: Stichting Afasie Nederland.

Fisher *Statistical Methods for Research Workers*, Edinburgh: Oliver and Boyd.

Hermens, H.J., Freriks, B., Merletti, R., Stegeman, D., Blok, J., Rau, G., Disselhorst-Klug, C. and Hagg, G. *European Recommendations for Suface ElectroMyoGraphy, results of the SENIAM project*, 2nd edn. Roessingh Research and Development b.v. 1999
